# Influence of lighting on sleep behaviour, circadian rhythm and spontaneous blink rate in stabled riding school horses (*Equus caballus)*

**DOI:** 10.1371/journal.pone.0326567

**Published:** 2025-06-27

**Authors:** Linda Greening, Eilis Harkin, Panoraia Kyriazopoulou, Zoe Heppelthwaite, Francesca Aragona, John A. Browne, Andrew Hemmings, Jane M. Williams, Barbara A. Murphy

**Affiliations:** 1 Equine Department, Hartpury University, Gloucester, Gloucestershire, United Kingdom; 2 School of Agriculture and Food Science, University College Dublin, Belfield, Dublin, Ireland; 3 Equilume Ltd, Naas, Co. Kildare, Ireland; 4 Royal Agricultural University, Cirencester, Gloucestershire, United Kingdom; Pennsylvania State University, UNITED STATES OF AMERICA

## Abstract

Modern horse husbandry involves significant time spent indoors, often in suboptimal lighting conditions and with frequent night-time disturbances by humans for management purposes. The aim of this study was to investigate the influence of a customised light-emitting diode (LED) lighting system and a standard fluorescent lighting fixture on equine sleep behaviours, circadian rhythmicity and spontaneous blink rates in horses. Ten riding school horses experienced two stable lighting conditions for four weeks each in a cross-over study running from January to March, 2023. The treatment lighting consisted of an LED system that provided timed, blue-enriched white polychromatic light by day and dim red light at night, and control lighting was a fluorescent tube that was turned on and off manually morning and evening. During week 4 of each experimental period, spontaneous blink rate was recorded twice for 30 min, behaviour of horses in their stables was recorded continuously for 72 h, and hair samples for circadian clock gene analysis were collected at 4-h intervals for 52 h. No differences were detected for total sleep, lateral or sternal recumbency, wakefulness, standing, standing sleep, or spontaneous blink rate (P > 0.05), between lighting conditions. The lighting period (Day versus Night) influenced total sleep (P < 0.01), total recumbency (P < 0.01), wakefulness (P < 0.01), and standing sleep (P < 0.05) in both conditions. For the treatment condition only, higher wakefulness was recorded during Day (P < 0.05). An overall effect of time for clock genes *PER2* and *DBP* was detected (P < 0.01), but there was no effect of treatment, or time by treatment interaction. Cosinor analysis detected significant 24-h rhythmicity for *PER2* and *DBP* (P < 0.01) in both lighting conditions. Results imply that dim red light at night does not negatively impact normal sleep patterns or circadian rhythmicity, and provide evidence supporting further research to better understand the role of blue-enriched LED light at promoting increased wakefulness during daytime in stabled horses.

## Introduction

Equestrianism is popular worldwide, with millions of horses and riders participating in competitive horse sports and non-competitive leisure riding [1]. Horse riders, owners and trainers are responsible for the management of their horses, with a duty of care to engage in practices which optimise equine health and welfare [2]. Horses (*Equus* *Caballus*) used in equestrian sport are often stabled as part of their normal management regimes, with up to 36% stabled all year round in the UK according to the recent British Equestrian Trade Association survey [3], despite evidence that stabling can compromise equine welfare by increasing stereotypic and abnormal repetitive behaviours [4]. Similarly, stable lighting regimes as well as bedding depth are known to influence sleep behaviour and quality, with less time spent in recumbent sleep stages on sub-optimal bedding [5] and sleep disturbances associated with nighttime light exposure [6]. Sleep is critical to the well-being of all animals and is co-regulated via circadian and homeostatic mechanisms [7]. It is affected by a range of environmental factors in horses [8], whereas light is the primary stimulus affecting sleep in humans [9,10]. However, the effects of disrupted sleep on welfare are currently poorly understood by both animal welfare scientists [11] and horse owners alike [12].

The circadian rhythm is an endogenous 24-hour cycle regulating physiology and behaviour entrained by external stimuli [13], primarily by the daily alternating periods of light and dark [14]. The retina receives light signals and translates them into neural signals that travel along the retino-hypothalamic tract to the suprachiasmatic nucleus (SCN) [15]. The SCN, the master mammalian circadian clock located in the hypothalamus [16], synchronises the body’s internal functions with the external environment via neural and hormonal signals that in turn signal time-of-day messages to peripheral clocks located throughout the body [17,18]. Circadian synchronisation drives self-sustained mammalian circadian rhythms of rest-activity cycles, hormone secretion, cardiovascular activity, metabolism, immune function, alertness, and musculoskeletal performance [17–20]. Tissue-specific gene expression patterns align organ function with the environment [21], such that circadian measurements can be taken from almost all peripheral tissues [22]. The use of hair follicle cells provides a practical, non-invasive means of assessing the internal circadian clock phase, and the strength of circadian synchronisation with the environment [19,23]. Cyclical expression of clock genes *NR1D2* (Nuclear receptor subfamily 1, group D, member 2), *Per2* (Period homolog 2), and the clock-controlled gene *Dbp* (D-site of albumin promoter binding protein) have successfully been confirmed in both human and horse hair follicles [23,24].

As a primary mediator of the circadian signal throughout the body, the hormone melatonin is also responsible for regulating the sleep-wake cycle [25]. Light signals processed by the SCN signal the pineal gland to produce melatonin during the hours of darkness [26]. Melatonin rhythmically fluctuates between low circulating levels by day and high levels during darkness hours [27]. As well as its important role in transmitting and maintaining circadian and circannual messages, melatonin is an important health regulator, functioning to optimize night-time rest [28] and immune function [29]. Therefore, consistent sleep-wake cycles and healthy functioning of the circadian system requires the absence of light at night to facilitate the normal rise in melatonin production [30].

The circadian system and melatonin suppression are not equally affected by all types of light. The intrinsically photosensitive retinal ganglion cells within the retina that are responsible for regulating the master circadian clock are maximally stimulated by short wavelength blue light (465–485 nm) [31]. Circadian disruption from exposure to sub-optimal light spectra and intensities by day, as well as erratic exposure to light at night, is responsible for a wide array of human health conditions [32,33]. Intensely managed horses often spend a high proportion of the 24 h period stabled and can miss optimal daytime light intensities or the natural fluctuations in the environmental light-dark cycle. Anecdotally, incandescent bulbs and fluorescent tubes are more commonly used on equine establishments and predominantly emit light with peaks within the longer wavelength, orange/red region of the spectrum, that are not ideal for optimizing circadian rhythms [34]. Previous studies in horses showed that low-intensity blue light (465nm) optimally suppressed melatonin [35] and stable lighting incorporating blue-enriched polychromatic light by day provided a stronger entrainment cue to the circadian system [36].

Furthermore, equine management frequently necessitates interaction with horses at night, resulting in abrupt exposures to light at inappropriate times in the 24 h cycle. Exposure to bright light at night very strongly suppresses melatonin secretion in humans and other animals [28,37]. A similar acute suppressive affect has been observed for horses [35], with evidence of disruption to the sleep-wake cycle for stabled horses caused by different lighting regimes [5,36] and an immediate drop in plasma melatonin concentrations was shown to accompany the switching on of stable lights at night [38]. Conversely, low intensity red light does not disrupt the nightly melatonin rise in horses [39] and human studies have shown that red light, with wavelengths above 620 nm, can be used to permit night-time visibility, yet minimize circadian disruption and maintain melatonin production [40,41]. In rodents, white light intensities as low as 5 lux have been shown to disrupt the sleep-wake cycle [42], whereas <10 lux of red light facilitated normal rest-activity behaviour and metabolic function [43,44]. These findings led to the development of a stable lighting system incorporating blue-enriched polychromatic white light by day and red light at night [36], but the impact of this lighting system on sleep behaviour in horses has not yet been investigated. Finally, perturbations in sleep homeostasis have previously been linked to activation of stress pathways such as the hypothalamic-pituitary-adrenal axis (HPAA), in a range of mammalian species (see [45] for recent review). Moreover, cortisol as a primary endocrine mediator of the stress response has been positively correlated with spontaneous blink rate (SBR) in the horse [46]. In this regard, SBR has been used as a robust stress indicator in horses subjected to sudden arrival of a novel object [47] and sham clipping [48]. Therefore, SBR was utilised in this investigation, to gauge stress levels in animals subjected to both LED and fluorescent tube lighting systems.

Due to the meaningful impact of light on daily biological functioning, it is increasingly important to understand the consequences of lighting regimes on equine circadian regulation, sleep and arousal levels. This study therefore compared the impact of two lighting systems on sleep behaviour, circadian rhythm and spontaneous blink rates in stabled riding school horses. We hypothesized that these parameters would be impacted in horses under LED lighting providing timed exposure to blue-enriched light by day and dim red light at night compared to a standard manual lighting regime utilising fluorescent light fixtures.

## Methods

A crossover study design was used to investigate circadian clock gene expression in hair follicles, behavioural parameters related to sleep and wakefulness, and spontaneous blink rates in stabled horses under two lighting conditions: a customised Light-emitting diode (LED) lighting system (Equilume Stable Light; Equilume Ltd, Co. Kildare, Ireland; www.equilume.com) (Treatment) and a standard fluorescent lighting fixture (Control). The experiment involved behavioural observations of video footage, collection of hair follicle samples, as previously described [23,36], and observations of spontaneous blink rate (SBR). All procedures were described under USDA Category B. The study was conducted in an equestrian stabling facility at Hartpury University, Gloucestershire, United Kingdom, latitude 51.91012°, longitude −2.30609°. The Ethics Committee of Hartpury University granted ethical approval for the study with grant approval number ETHICS2021-63.

### Animals

Ten horses aged between five and seventeen years of mixed breed (riding horse/cob types), height, and sex (n = 7 geldings; n = 3 mares) were recruited through convenience sampling from a population of riding horses resident at Hartpury Equestrian Centre. All horses were acclimatised to their individual stables, measuring 3.6 m x 3.6 m and arranged in an American barn with a walkway in the centre, for one month prior to study commencement. The stables were partitioned by wooden panels with metal bars at the front and an open-top stable door facing into the central aisle. All horses were bedded on wood shavings (depth ~10 cm) covering approximately half of the stable floor, with rubber matting beneath covering the full floor. The stables were cleaned each morning between 07:00–08:00 and intermittently throughout the day until 19:00. Horses were rugged overnight according to individual needs. Horses had ad-lib access to water provided in a bucket and refreshed regularly throughout the day. One of the horses was fed twice daily with 2.5 kg of concentrated feed (Baileys Horse Feeds) in addition to ad-lib hay. The diets of the remaining nine horses consisted entirely of hay, which was provided ad-lib, so they did not exhaust their supply. No horses displayed signs of stereotypy, and all horses were deemed fit for exercise. The horses were stabled continuously but had a daily exercise and management period during which they were out of the stable for one to two and a half hours. The exercise timing (period out of the stable) occurred between 09:00 and 18:00 and varied per horse and day throughout the study.

### Study design

**Fig 1:**
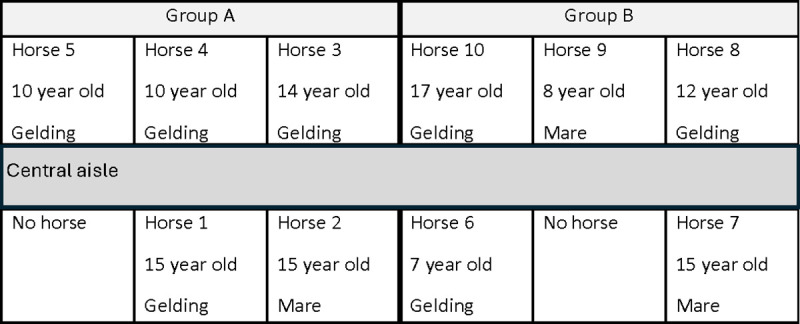
Schematic of the experimental barn layout and horse assignment to individual stables and groups.

Horses were randomly allocated to individual stables by the yard manager and then assigned to one of two groups A (mean age = 11.2 ± 4.1 years) or B (mean age = 12 ± 5 years) according to their location in the barn (Fig 1).

**Fig 2:**
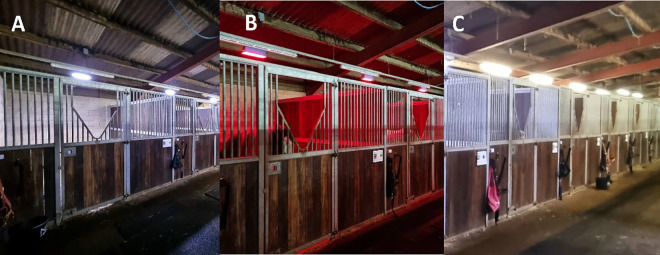
Images of the stable lighting. **(A)** Treatment nighttime (Equilume® Stable Light), **(B)** Treatment daytime (Equilume® Stable Light), and **(C)** Control daytime (fluorescent strip light).

**Fig 3:**
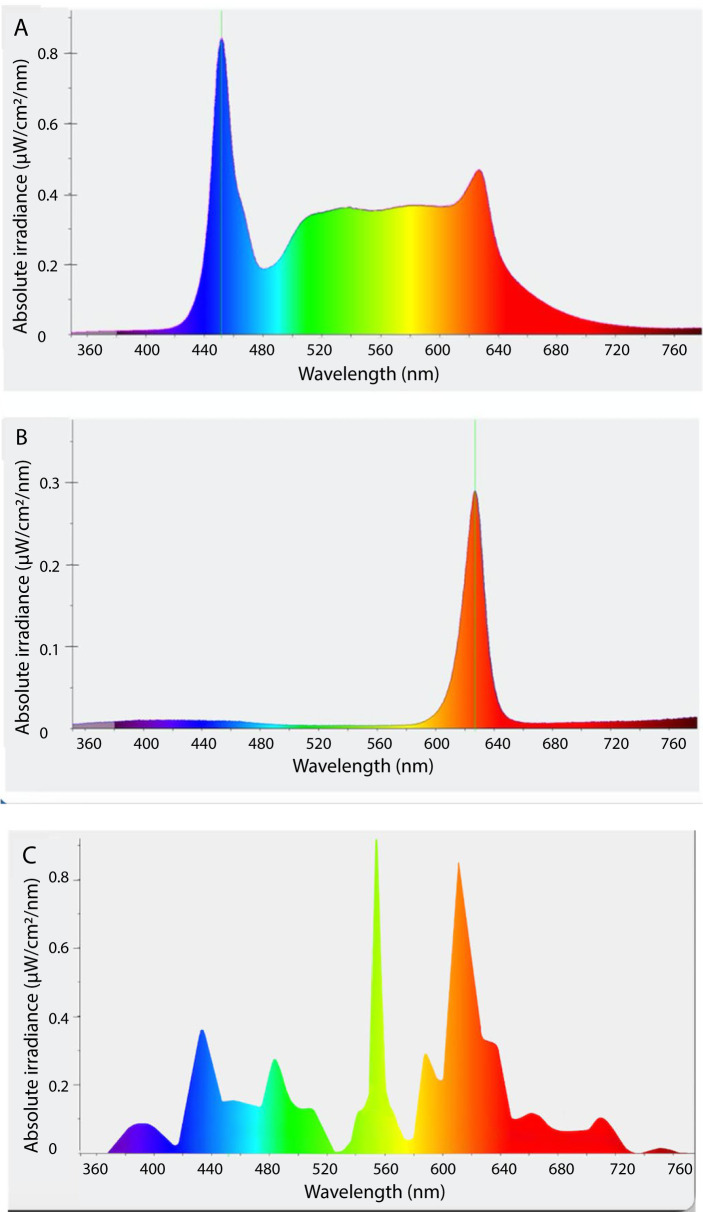
Spectral light profiles. **(A)** Blue-enriched white polychromatic light by day, (B) red light by night and (C) fluorescent light. Light measurements were taken using a CRI Illuminance Meter CL-70F at horse-eye level, with the light sensor facing upwards.

Each individual stable had both a Treatment and Control lighting fixture fitted, as follows: Treatment lighting comprised an Equilume® Stable Light (Equilume Ltd, Kildare, Ireland) that was secured 3.5 m above ground level and central to each stable, providing an approximate light coverage of 4.5 x 4.5 x 3.5 m [49]. These lights provided blue-enriched white polychromatic light by day (peak wavelength 460 nm and mean light intensity 487.8 ± 41.5 lux) and dim red light (peak wavelength 620 nm and mean light intensity 9.7 ± 0.2 lux) at night. Lights transitioned gradually over 20 min from red to white at dawn (07:20) and white to red at dusk (20:20) to give approximately 13 h of daytime light when in use (Fig 2). Control lighting consisted of a 5 ft T8 fluorescent 58W 3000K tube light (Bell, Normanton, West Yorkshire, UK) secured 3.8 m above ground level and placed centrally in each stable, providing an approximate light coverage of 4 x 5 x 3 m, (peak wavelength 560nm and mean light intensity of 227.8 ± 7.5 lux). When in use, these were manually turned on each morning between 07:00 and 07:30 (no transition) and turned off each night between 20:00 and 21:00 (no transition), providing approximately 13–14 h of daytime light and darkness conditions at night. Spectral light profiles from measurements taken at horse eye level using a CRI Illuminance Meter CL-70F are presented in Fig 3 and light intensity readings within each stable under each lighting condition (Treatment and Control) are detailed in Table 1.

**Table 1:** Light intensity measurements (lux) captured during Day and Night from each stable using a CRI Illuminance Meter CL-70F when positioned centrally with the light sensor facing upwards at horse eye level height.

Animal	Treatment		Control	
	Day (lux)	Night (lux)	Day (lux)	Night (lux)
Horse 1	368	10	234	<1
Horse 2	484	9.25	213	<1
Horse 3	575	11	212.5	<1
Horse 4	529.5	10.2	243	<1
Horse 5	552.5	8.6	266	<1
Horse 6	363.5	9.8	230.5	<1
Horse 7	401.5	10	248.25	<1
Horse 8	788	9.3	242.5	<1
Horse 9	430.5	10.1	194	<1
Horse 10	385	9	194.5	<1
**Mean±SEM**	487.8 ± 41.5	9.7 ± 0.2	227.8 ± 7.5	<1

Treatment and Control lighting systems were set up on different electrical circuits such that a single lighting system could be active within an individual stable at a time to facilitate the different experimental phases of the study. The stables did not have floor-to-ceiling partitions such that a slight spillover of light was visible in stables either side of the Group A/ Group B. However, this spillover did not impact the light intensities recorded for Day or Night within lighting condition as the intensity values recorded in stables either side of the treatment divide fell within the range for that group and did not vary from the group mean (Table 1).

### Lighting treatment periods

Horses experienced each lighting condition once over two four-week experimental periods. The first experimental period took place between January 23^rd^ and February 18^th^, 2023, during which time Group A experienced the treatment lighting condition and Group B experienced the control lighting condition. The second four-week period took place between February 19^th^ and March 19^th^ 2023, when the lighting conditions for each group were switched. On week four of each experimental period, video footage of each horse in its stable was recorded continuously on days 1–3 for 72 h, hair sample collections were carried out on Days 4–5 at four-hour intervals starting at 08:00 for 52 h, and SBR recording occurred within a two-hour window from 17:00–19:00 on Days 1 and 3 for all horses (Table 2).

**Table 2:** The data collection schedule for video recording, hair sample collection and spontaneous blink rate (SBR) recording during week four of each experimental period.

Video Recording			Hair Follicle Collection														
**1**	**2**	**3**	**4**						**5**						**6**		
**Monday**	**Tuesday**	**Wednesday**	**Thursday**						**Friday**						**Saturday**		
Video Recording commenced at 0:00 for 72 hours. SBR recording between 17:00 and 19:00	Video Recording.	Video recording concluded at 0:00. SBR recording between 17:00 and 19:00	Hair follicle collection commenced at 08:00 at 4-hour intervals for 52 hours.						Hair follicle collection.						Hair follicle collection concluded at 08:00.		
**0:00**	**0:00**	**0:00**	**0:00**	**04:00**	**08:00**	**12:00**	**16:00**	**20:00**	**0:00**	**4:00**	**8:00**	**12:00**	**16:00**	**20:00**	**0:00**	**04:00**	**08:00**

### Behavioural data collection

#### Spontaneous blink rate.

Spontaneous blink rate data was measured by securely attaching Go-Pro Hero 10 cameras (7 x 5 x 4 cm Go-Pro Incorporated) to the headcollars of each horse individually using Same Top^TM^ backpack mount attachments (8.5 x 5 x 4.3 cm, model number SA-YG16). Cameras were set up to record footage at a quality of 4k/60FPS at 1x speed. The camera was pointed at the left eye avoiding the animals’ direct line of vision or contact with the eye [47]. Where necessary, horse’s forelocks were plaited to avoid obstruction to the lens of the camera. Measurements were taken between 17:00 and 19:00, a normally quiet period for the yard and prior to the final evening forage ration arriving. Five cameras were used to simultaneously record SBR from Group A over 30 min, and then swapped to Group B horses for 30 min. A period of ten minutes was allowed to enable horses to acclimatise to the equipment and the presence of the researchers prior to recording. Recordings occurred twice for each horse on non-consecutive days to facilitate the calculation of a mean value for each individual and eye blink behaviour measured [50]. Horses were loosely tied via a lead-rope attached to a headcollar to a tie ring at the front of the stable for the duration of the recording and observed from a distance to ensure that cameras remained in position. Finally, full eye blinks, defined as momentary but full closure of the eyelid [51] were quantified by a single trained observer using a mechanical tally counter (Tebery Products^TM^) from the gathered video footage.

#### Sleep behaviour.

**Fig 4:**
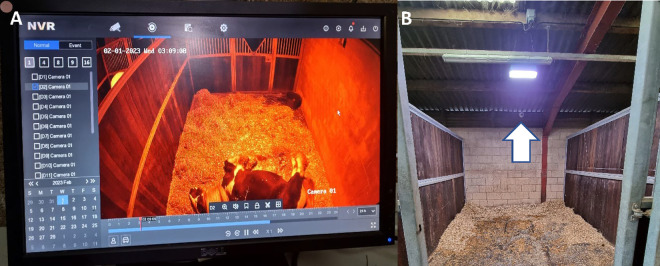
Video recording within stables. **(A)** Network Video Recorder visual of a stable and (B) placement of camera (denoted by white arrow) within the stable.

Behavioural observations of horses within stables were recorded for consecutive 24-h periods over three consecutive days in each experimental condition (Table 2) using focal continuous sampling against a predefined ethogram (Table 3) and Hikivision CCTV equipment comprising a 16 channel 4K Power over Ethernet (PoE) Network Video Recorder (NVR) (Fig 4A) and 4MP Acusense fixed lens (2.8/4 mm) cameras (one per stable, Fig 4B) with 30 m infrared range. Cameras were installed in stables via network cables secured at a height that prevented horses from interfering with them. Footage was downloaded from all cameras at the end of the recording period onto a Seagate Portable 2TB, External Hard Drive. All files were then uploaded to a secure SharePoint site on MS OneDrive.

**Table 3:** Ethogram describing (sleep) behavioural states for horses (adapted from [5]).

Sleep State	Definition
Wakefulness (awake)	Horse is ingesting, drinking, excreting or moving, characterised by frequent ear movement and eyes open/blinking.
Standing (awake)	Horse is immobile but there may be readjustment of limbs, itching, chewing licking etc. With or without resting hindlimb. The head is low/ close to vertical but poll will be above withers. Eyes open/half shut or frequent opening and shutting. Ear movement occurs but less frequently.
Standing (sleep)	After a distinct head drop where the poll is equal to or lower than the withers, the horse is standing immobile. Eye partially or completely shut and low frequency, if any, blinking. Little if any ear movement (ears not pointing forward). With or without resting hindlimb.
Sternal recumbency (sleep)	Horse is lying down, sternum in contact with ground, legs folded beneath. Head and limbs are immobile, eye partially or completely shut/ low frequency, if any, blinking. Little if any ear movement (ears not pointing forward).
Sternal recumbency (awake)	Horse is lying down, sternum in contact with ground, legs folded beneath. Large movements such as scratching or eating are observed as a state of wakefulness, further characterised by frequent ear movement and eyes open/blinking.
Lateral Recumbency (sleep)	Horse is lying on its side, legs extended, head on floor. Horse is largely immobile although there may be (limb/body) twitches/ some ear movement.
Out of the stable (awake)	The time the horse leaves the stable to the time it returns.
Unknown	At any point during observation when the horses head moves out of view, i.e., we do not know if they are in a state of wakefulness of sleep.

### Hair follicle collection for clock gene expression

Mane hair samples, complete with follicles, were collected from each horse at 4 h intervals for 52 h from 08:00 on day four of week four in each experimental period. Each sample comprised 10–20 hair which were carefully trimmed and placed in 2 ml tubes containing 0.75 ml of RNAlater® solution. Overnight sampling was conducted using red light headlights and torches. Samples were stored at ambient temperature (<18°C) while in transit for 48 h to the laboratory, conditions shown not to compromise RNA quality or quantity in hair follicle samples stored in RNAlater® [52]. On arrival, the samples were stored at 4°C for 72 h before being decanted and stored at −80°C until analysis.

#### RNA isolation.

Following removal from the freezer, the hair samples were further trimmed to just above the hair follicles and transferred to a nuclease-free microcentrifuge tube using forceps. RNA was isolated using the Quick-RNA Microprep Kit (Zymo Research, California, USA) as per the manufacturer’s instructions with the following modifications: a single 5 mm steel bead was added to each tube before adding 500µl of RNA Lysis Buffer. Following the addition of the Lysis Buffer the hair follicle samples were homogenized for two minutes at a frequency of 30 hertz using the Qiagen TissueLyser system. All remaining steps were as per the supplier’s instructions except for the inclusion of a final two minute centrifugation step to ensure complete removal of any residual wash buffer. The RNA was eluted in 15 µl and stored at −80°C. RNA quantity was measured using the NanoDrop Spectrophotometer (Thermo Fisher Scientific, MA, USA) and RNA quality was assessed using the Agilent Bioanalyzer RNA 6000 (Agilent Technologies, CA, USA).

#### Quantitative polymerase chain reaction (qPCR).

For each sample, 250 ng RNA was converted to complementary DNA (cDNA) in a 20 μl reaction using the Applied Biosystems High-Capacity cDNA Reverse Transcription Kit (Thermo Fisher) as per the manufacturer’s instructions. A cDNA pool containing 3 μl from each sample was prepared and used to generate a 7-point, 1 in 4 serial dilutions for the standard curve, positive controls and inter-plate calibrators. Quantitative polymerase chain reaction (qPCR) was performed to detect the relative expression *NR1D2* (Nuclear receptor subfamily1, group D, member 2), *PER2* (Period homolog 2) and the clock-controlled gene *DBP* (D-site of albumin promoter binding protein) using the Applied Biosystems 7500 FAST Sequence Detection system (Thermo Fisher Scientific, MA, USA) and SYBR green chemistry (Roche, Burgess Hill, UK). These candidate genes were selected based on prior research indicating that they are expressed cyclically in human and horsehair follicles [23,24]. Primer design was performed using the Primer BLAST tool (https://www.ncbi.nlm.nih.gov/tools/primer-blast/) based on the equine genome sequence assembly available in the NCBI database https://www.ncbi.nlm.nih.gov/datasets/genome/GCF_002863925.1/, version EqCab3 GCA_002863925.1 All primer sequences are provided in S1 Table. The efficiencies of all primers used in the study were determined using the standard curve method and were shown to lie between 90% and 110%. The stability of eight potential reference genes was assessed for suitability as internal controls using the geNORM function within the qBASE+ (Ghent, Belgium) analysis software. *PPIA* (Peptidylprolyl Isomerase A) and *RPL19* (ribosomal protein L19) were shown to be the most stably expressed, with a geNorm M value >0.2 indicating very high reference gene stability and were selected to normalise the gene expression data. The qPCR reactions were prepared in duplicate in a 20µl reaction as per the manufacturer’s instructions (FastStart Universal SYBR Green Master, Roche) using a final concentration of 300 nM for each primer. The thermal cycling involved one cycle at 50°C for two minutes, followed by one cycle at 95°C for 10 minutes and 40 cycles at 95°C for 15 sec, ending with 60°C for one minute.

### Data analysis

#### Spontaneous blink rate.

Analysis via Shapiro-Wilk’s test indicated that the data sets were normally distributed. Paired t-tests were used to compare mean values of full blinks under both lighting systems (GraphPad Prism version 10.3.1 for Windows [GraphPad Software, Boston, Massachusetts USA, www.graphpad.com]). Data was presented as means ± SEM and P < 0.05 was considered significant.

#### Sleep behaviour.

In total, 1,440 hours of footage were recorded. To facilitate analysis (one hour of footage = 10–20 minutes to review), ten observers were trained on use of the ethogram and data recording using an Excel spreadsheet by the lead author. This comprised three meetings to 1) explain and refine the ethogram, 2) instruct on correct recording of observations from sample videos using a pre-prepared template spreadsheet, and 3) to clarify any remaining issues following completion of recording behaviours from sample videos used for completion of inter-observer reliability testing. Subsequently, each observer was assigned all the footage for one horse. Before data analysis, the lead author checked that the data for each horse on each of the recorded days totalled 24 h (+/- 60 sec) and reviewed the video footage to make corrections where necessary.

Intra-class correlation coefficient (ICC) tests conducted to determine the inter-observer reliability amongst the 10 observers prior to reviewing the experimental footage showed a high-reliability rating of 0.92 (ICC absolute agreement for single measures F = 88.6; P < 0.01). Results of inter-observer reliability tests supported the consistency of the observed behaviour reporting from the ten observers.

Total sleep duration was calculated as the summation of ‘lateral recumbency (sleep)’, ‘sternal recumbency (sleep)’ and ‘standing (sleep)’ data combined; total recumbency duration was calculated by the summation of ‘lateral recumbency (sleep)’, ‘sternal recumbency (sleep)’ and ‘sternal recumbency (awake)’ data combined. In addition, ‘sternal recumbency (sleep and awake)’, ‘lateral recumbency (sleep)’, ‘wakefulness’, ‘standing (awake)’, ‘out of-stable’ and ‘unknown’ behaviour durations were analysed individually. All behaviour data underwent statistical testing using GraphPad Prism version 10.3.1 for Windows. All data were first assessed for normality and lognormality using D’Agostino & Pearson tests. Statistical differences between lighting conditions (Treatment versus Control) and between lighting periods (‘Day versus ‘Night’) were assessed using paired t-tests or Wilcoxon tests where data was shown to be normally or not normally distributed, respectively. The lighting period ‘Day’ was defined as behaviours that occurred between 07:20–20:20 during the experimental observation periods. The lighting period ‘Night’ was defined as behaviours that occurred between 20:21 and 07:19 during the experimental observation periods.

One-way repeated measures ANOVA or a Friedman’s test was conducted to determine the impact of the lighting period (Day or Night) on the horses’ behaviour depending on data normality followed by Tukey’s or Dunn’s multiple comparisons tests, where appropriate, to examine differences between and within each lighting condition during each lighting period. These tests assessed differences across the four groups: Control Night, Control Day, Treatment Night and Treatment Day. Data were considered statistically significant if P < 0.05.

#### Hair follicle clock gene expression.

Data from hair follicle samples for all 10 horses under each lighting condition were assessed. Calibrated Normalized Relative Quantities (CNRQ) were calculated for each sample and each gene transcript using the qBASE+ software package (Ghent, Belgium) and the two reference genes RPL19 and PPIA. The data were normalised within horse to account for individual clock gene expression abundance differences. Repeated measures two-way ANOVA was used to assess the effect of time, treatment, and time x treatment interaction on gene expression in all horses (n = 10) (GraphPad Prism version 10.3.1 for Windows). Data were considered statistically significant if P < 0.05 and were presented as means ± S.E.M. Where repeated measures two-way ANOVA found significant differences in the effect of time, cosinor analysis [53] based on the least squares cosine fit method [54] was used to assess the presence of 24 h (circadian) periodic variance. This cosinor method produced an estimate of acrophase (time of peak value) and robustness (stability).

## Results

### Spontaneous blink rate

**Fig 5:**
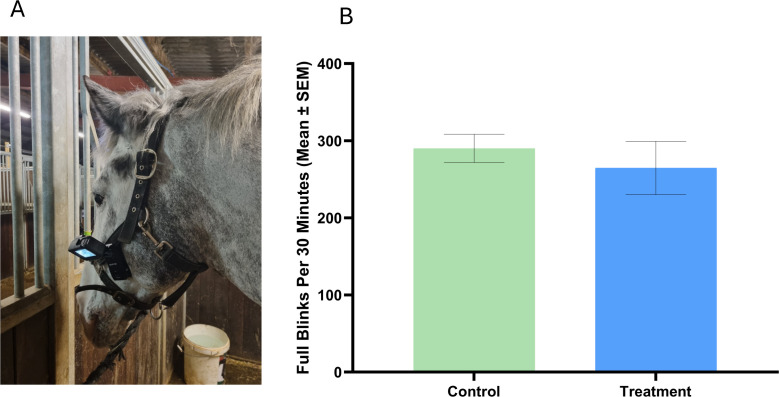
Spontaneous blink rate. **(A)** Example of Go-Pro Hero 10 camera (7 x 5 x 4 cm) attached to the headcollar of a horse for collection of spontaneous blink rate data. **(B)** Comparison of Full Blinks between Fluorescent (control) and LED (treatment) lighting conditions presented as means ± SEM.

No differences were detected for full blink rate between control (290 ± 18.32) and treatment (264.7 ± 34.61) lighting conditions (P > 0.05; Fig 5B).

### Sleep behavioural data

No differences were observed in the mean duration of each behavioural parameter exhibited between Treatment or Control lighting conditions (P > 0.05). Day versus Night differences existed for each behavioural parameter regardless of lighting condition (P < 0.05) with the exception of Standing (awake) and Unknown (P > 0.05) (Table 4).

**Table 4:** Comparison of behaviour patterns between Control and Treatment lighting conditions and between Day and Night.

	LED Lighting Treatment			Lighting Period		
	Control (n = 10)	Treatment (n = 10)	P-value	Day (n = 10)	Night (n = 10)	P-value
Behavioural parameter	Duration in sec (duration in h) Mean ± SEM			Duration in sec (duration in h) Mean ± SEM		
Total Sleep^a^	10967 ± 2045 (3.04 ± 0.57)	10871 ± 1908 (3.02 ± 0.53)	0.945	1535 ± 428.9 (0.43 ± 0.12)	9421 ± 1526 (2.62 ± 0.42)	<0.001
Total Recumbency^b^	9441 ± 1406 (2.62 ± 0.39)	9245 ± 1357 (2.57 ± 0.38)	0.770	200.9 ± 52.58 (0.06 ± 0.01)	8713 ± 1320 (2.42 ± 0.37)	<0.001
Sternal Recumbency^c^	7343 ± 998.1 (2.04 ± 0.28)	7268 ± 992.8 (2.02 ± 0.28)	0.695	184.4 ± 49.45 (0.05 ± 0.01)	6795 ± 939.8 (1.89 ± 0.26)	<0.001
Lateral Recumbency	2098 ± 711.6 (0.58 ± 0.2)	1975 ± 651.9 (0.55 ± 0.18)	0.765	16.45 ± 11.02 (0.005 ± 0.003)	1918 ± 633.9 (0.53 ± 0.18)	0.016
Wakefulness	44692 ± 2076 (12.41 ± 0.58)	42949 ± 1344 (11.93 ± 0.37)	0.248	24679 ± 1013 (6.86 ± 0.28)	18191 ± 1118 (5.05 ± 0.31)	0.004
Standing (awake)	12453 ± 2120 (3.46 ± 0.59)	15433 ± 2288 (4.29 ± 0.64)	0.105	7229 ± 1395 (2.01 ± 0.39h)	7394 ± 850.5 (2.05 ± 0.24h)	0.870
Standing sleep	4809 ± 1610 (1.34 ± 0.45)	4415 ± 1235 (1.23 ± 0.43)	0.786	1455 ± 421.6 (0.40 ± 0.12)	3533 ± 887.9 (0.98 ± 0.27)	0.005
Out of stable	6050 ± 491.8 (1.68 ± 0.14)	6988 ± 407 (1.94 ± 0.11)	0.152	6471 ± 403.7 (1.80 ± 0.11)	0 ± 0 (0 ± 0)	N/A
Unknown	7530 ± 2460 (2.09 ± 0.68)	5885 ± 2199 (1.63 ± 0.61)	0.469	2288 ± 889.7 (0.64 ± 0.24)	4461 ± 1743 (1.24 ± 0.48)	0.065

P-value of <0.05 was considered significant.

Sec: seconds; h: hours; SEM: standard error of means;

^a^Total Sleep includes the following behaviours: Sternal Recumbency (Sleep), Lateral Recumbency (Sleep) and Standing (Sleep).

^b^Total Recumbency includes the following behaviours: Sternal Recumbency (Sleep), Sternal Recumbency (Awake) and Lateral Recumbency (Sleep).

^c^Sternal Recumbency includes the following behaviours: Sternal Recumbency (Sleep), Sternal Recumbency (Awake).

* Not Applicable (N/A); The horses were not out of the stable during the night.

### Total sleep

**Fig 6:**
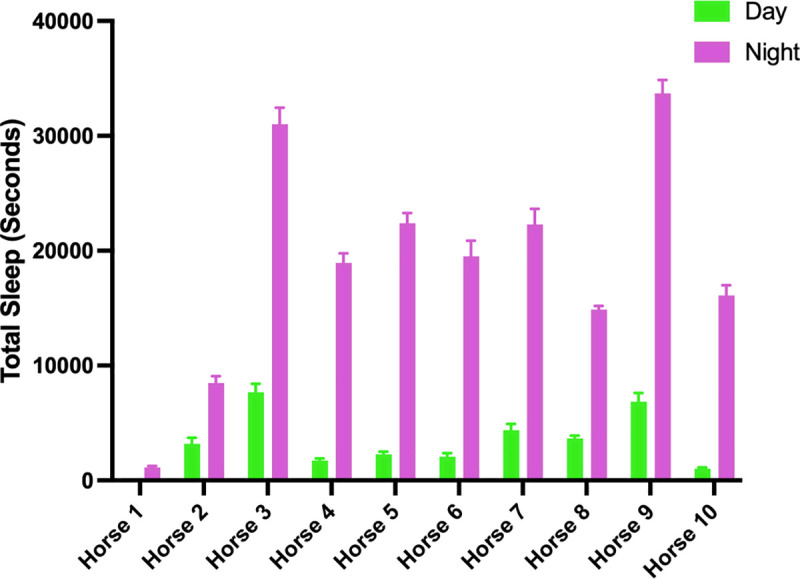
Total sleep duration during Day and Night for three consecutive 24 h periods for each individual horse. Data are presented as means ± SEM.

Data for total sleep duration (comprising lateral recumbency, sternal recumbency sleep, and standing sleep) were found to be normally distributed. No overall effect of treatment conditions was observed (P = 0.945; Table 4) with a mean total sleep duration of 3.04 h for horses under the control lighting condition and 3.02 h under the treatment lighting condition. Data for the lighting period (Day and Night) were normally distributed. The lighting period affected total sleep duration, with higher values observed during the night (P < 0.001; Table 4). Multiple comparison tests revealed differences within each lighting condition for Day vs Night (P < 0.001: Table 5) but no differences were observed between Control Day and Treatment Day (P = 0.333) or Control Night and Treatment Night (P = 0.995). Horses displayed inter-individual variation in behavioural profiles for sleep (Fig 6).

**Table 5:** Multiple comparisons analyses of the behaviour patterns total sleep, total recumbency and wakefulness for Control and Treatment conditions by the Lighting Period (Day and Night).

Total Sleep duration^a^ (sec)							
	Mean	SEM	P-value	Multiple Comparisons P-value			
				Control Day	Treatment Day	Control Night	Treatment Night
Control Day	2065	421.3	<0.001	–	0.333	0.003	0.003
Treatment Day	1205	490.3		0.333	–	>0.001	>0.001
Control Night	9299	1624		0.003	<0.001	–	0.995
Treatment Night	9545	1596		0.003	<0.001	0.995	–
**Total recumbency duration**^b^ **(sec)**							
	Mean	SEM	P-value	Multiple Comparisons P-value			
				Control Day	Treatment Day	Control Night	Treatment Night
Control Day	458.5	244.1	<0.001	–	>0.999	0.034	0.019
Treatment Day	18.15	17.87		>0.9999	–	0.002	>0.001
Control Night	5759	1109		0.0335	0.002	–	>0.9999
Treatment Night	6023	987.8		0.0194	0.001	>0.9999	–
**Wakefulness duration (sec)**							
	Mean	SEM	P-value	Multiple Comparisons P-value			
				Control Day	Treatment Day	Control Night	Treatment Night
Control Day	23348	1995	0.007	–	0.998	0.314	0.044
Treatment Day	23736	1218		0.998	–	0.247	0.028
Control Night	19316	1640		0.314	0.247	–	0.605
Treatment Night	17051	1186		0.044	0.028	0.605	–

P-value of < 0.05 was considered significant.

Sec: seconds; SEM: standard error of means.

^a^Total Sleep includes the following behaviours: Sternal Recumbency (Sleep), Lateral Recumbency (Sleep) and Standing (Sleep).

^b^Total Recumbency includes the following behaviours: Sternal Recumbency (Sleep), Sternal Recumbency (Awake) and Lateral Recumbency (Sleep).

### Total recumbency

**Fig 7:**
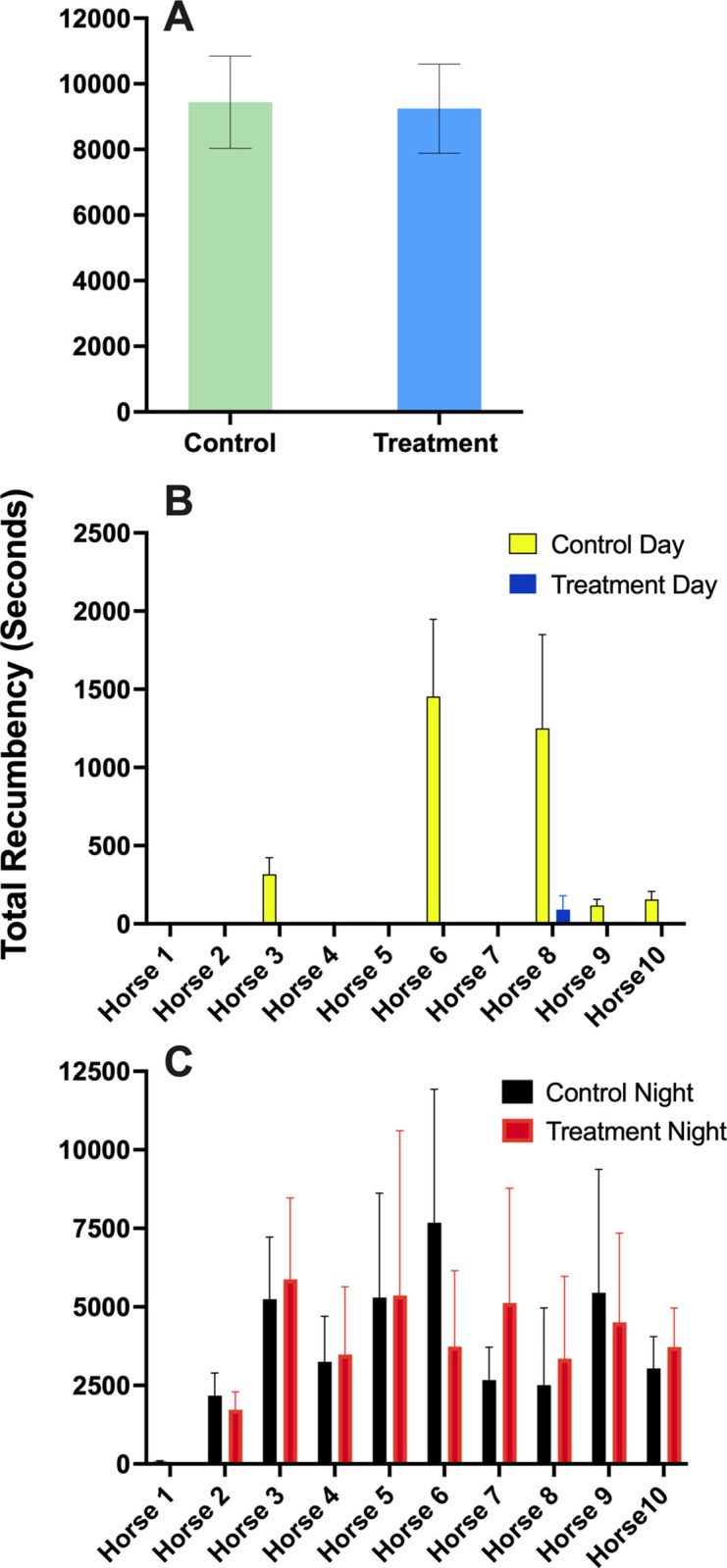
Total recumbency duration of horses maintained under customised LED lighting (treatment) and fluorescent lighting (control) for three consecutive days. (A) Total recumbency duration during Day (B) and Night (C) for each lighting condition (control and treatment). Data are presented as means ±SEM for three consecutive 24-h periods.

Data for total recumbency (comprising lateral recumbency, sternal recumbency sleep and sternal recumbency awake) were found to be not normally distributed. No overall effect of treatment condition was observed (P = 0.77; Table 4; Fig 7A). Data for the lighting period (Day vs Night) were normally distributed. The lighting period had an effect on total recumbency duration, with higher values observed during the night (P < 0.001; Table 4). Multiple comparison tests (Table 5) revealed differences within each lighting condition for Day vs Night (P < 0.001) but no differences were observed between Control Day and Treatment Day (P > 0.999) or Control Night and Treatment Night (P > 0.999; Fig 7B and 7C).

### Sternal and lateral recumbency

The data for sternal recumbency were found to be not normally distributed. No overall effect of treatment condition was observed (P = 0.695). The data for lateral recumbency were found to be not normally distributed. No overall effect of treatment condition was observed (P = 0.765). The lighting period had an effect on both sternal (P < 0.001) and lateral recumbency (P = 0.016) duration, with higher values observed during the night (Table 4). Multiple comparison tests revealed differences within each lighting condition for Day vs Night (P < 0.001) but again no differences were observed for either sternal or lateral recumbency durations between Control Day and Treatment Day or Control Night and Treatment Night (P > 0.999 for all, Table 5).

### Wakefulness

**Fig 8:**
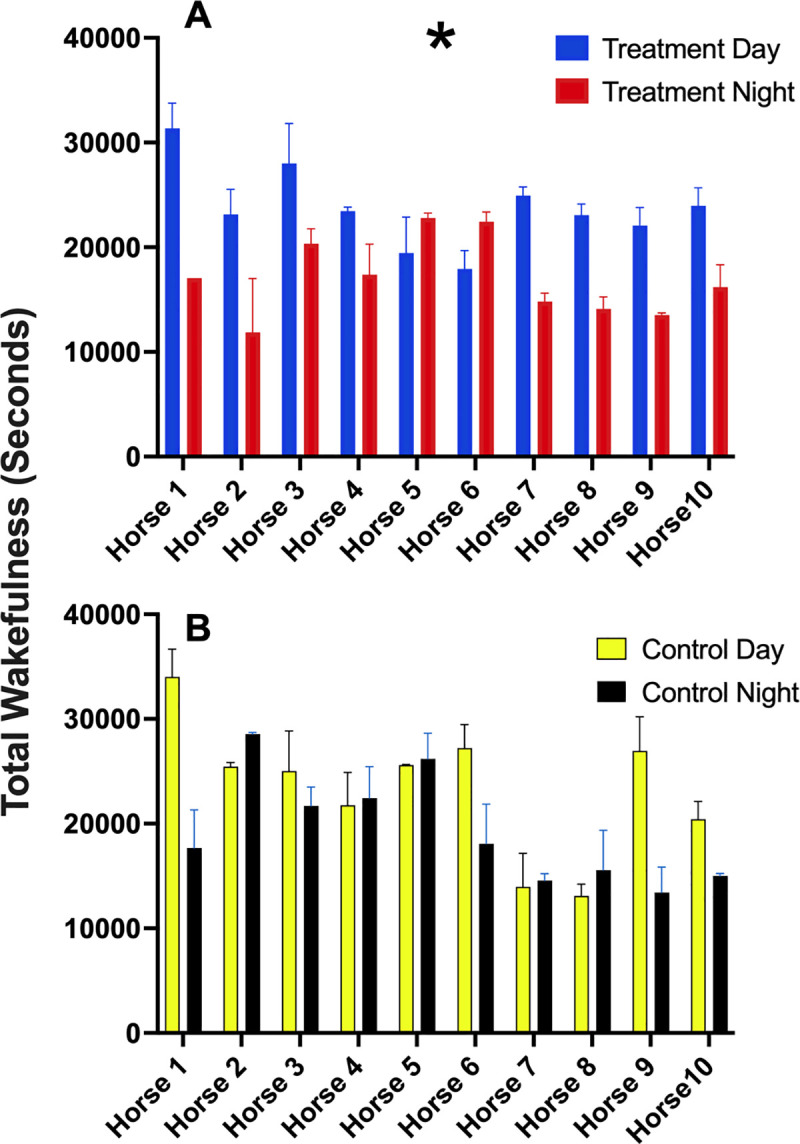
Day versus night durations in wakefulness for three consecutive days. **(A)** Day versus night duration under treatment (LED) and **B)** Day versus night duration under control (fluorescent) lighting conditions Data are presented as means + /-SEM for three consecutive 24 h periods. * indicates P < 0.05.

Data for wakefulness were found to be not normally distributed. No overall effect of treatment condition was observed (P = 0.248; Table 4). Data for lighting period (Day and Night) were normally distributed. The lighting period had an effect on wakefulness duration, with higher values observed during the day (P < 0.004; Table 4). Multiple comparison tests revealed Day vs Night differences (P = 0.007), with higher wakefulness duration by day for the treatment condition only (P = 0.028). No differences were observed for the control lighting condition for Day vs Night (P = 0.314; Table 5). No differences were observed between Control Day and Treatment Day (P = 0.998) or Control Night and Treatment Night (P = 0.605). Data for each horse during each lighting condition and lighting period are presented in Fig 8.

### Additional behaviour parameters

There were no differences in mean durations between lighting condition for the parameters standing (awake) (P = 0.105), standing sleep (P = 0.786), out of stable (P = 0.152) and unknown (P = 0.469; Table 4). No differences were observed between Day and Night for standing awake (P = 0.87) and unknown (P = 0.065), and higher values for standing sleep were observed during Night (P = 0.005; Table 5).

### Clock gene expression

**Fig 9:**
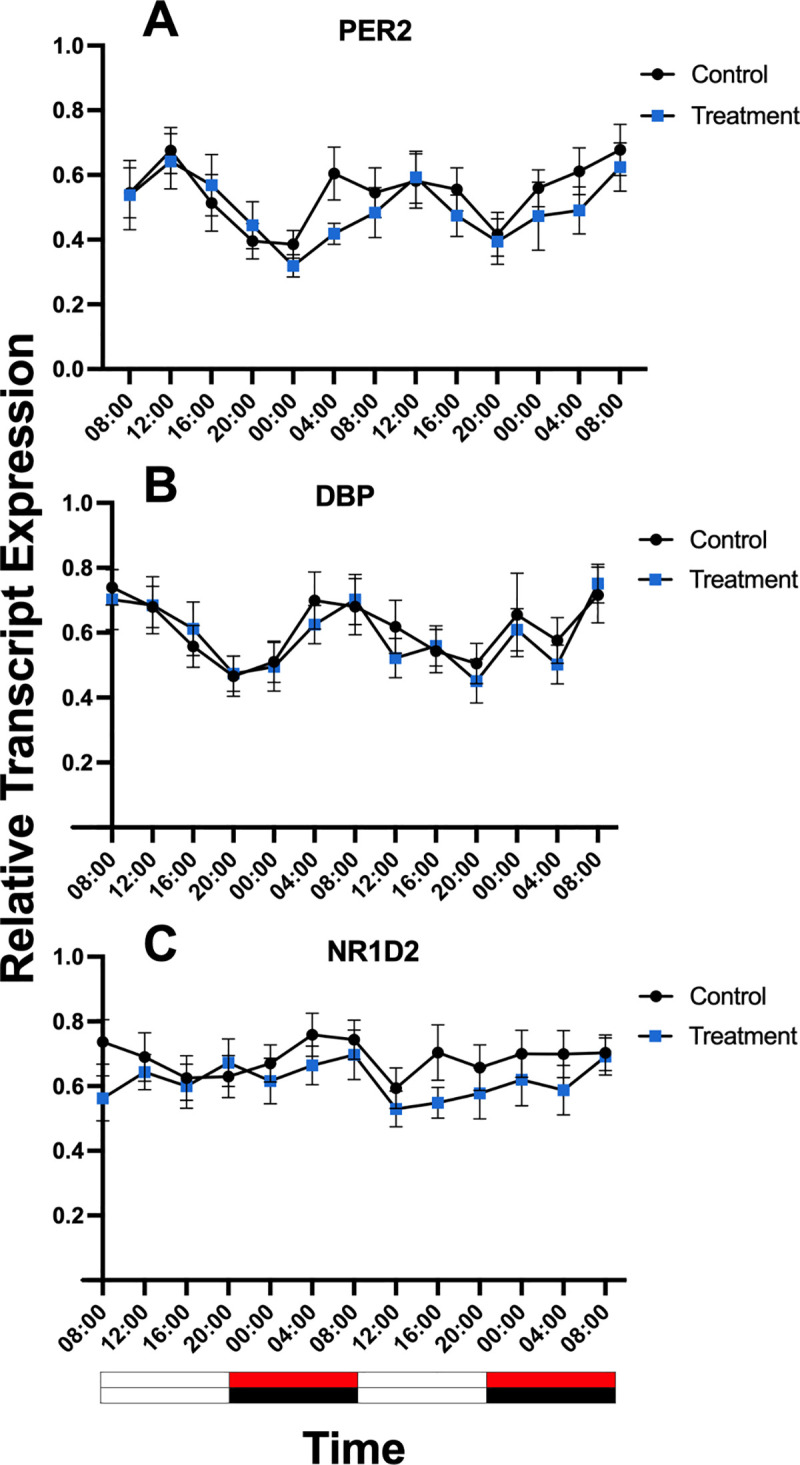
Profiles of clock gene expression in hair follicles from horses (n = 10) maintained under customised LED lighting (treatment) or fluorescent (control) lighting conditions. **(A)** *PER2*, **(B)** *DBP* and **C)** *NR1D2*. Data are presented as means ± SEM. There was a significant overall effect of time for *PER2* and *DBP* (P < 0.01; P < 0.001), but no effect of treatment or time by treatment interaction (P > 0.05). No effect of time, treatment or time by treatment interaction was detected for *NR1D2* (P > 0.05). White and red or black bars beneath the graphs represent periods of daytime light and red light or darkness at night, respectively.

Expression of the core clock genes *PER2, NR1D2*, and the clock-controlled gene *DBP* were detected in all samples (Fig 9). The data was normally distributed. There was an overall effect of time for *PER2* and *DBP* (P < 0.01; P < 0.001), but no effect of treatment or time by treatment interaction (P > 0.05). No effect of time, treatment or time by treatment interaction was detected for *NR1D2* (P > 0.05).

#### Cosinor analysis results.

Cosinor analysis detected 24-h rhythmicity for both *PER2* and *DBP* (P < 0.01) in control and treatment lighting conditions (Table 6).

**Table 6:** Cosinor analysis of 24-h clock gene expression profiles in hair follicles. Robustness (rhythm stability), acrophase (time of rhythm peak) and P-values are presented for horses maintained in treatment and control lighting conditions.

Gene Transcript	Robustness %		Acrophase (24-h)		P-Value	
Treatment	Control	Treatment	Control	Treatment	Control
*PER2*	68.8	57.5	11:24	08:58	0.00	0.01
*DBP*	74.1	57.0	08:53	07:47	0.01	0.00

## Discussion

Appropriate exposure to lighting that mimics the environmental 24 h cycle is considered a key factor in the correct functioning of the mammalian circadian system, which in turn confers improved rest/activity rhythms and ultimately benefits well-being. This study investigated the influence of a customised LED lighting system and standard fluorescent lighting fixtures on arousal, sleep behaviours and circadian rhythmicity in stabled horses for the first time. The results reveal no overall difference in mean durations of any parameter over 24-h measured in horses maintained under the two different lighting conditions. Under both conditions, sleep and wakefulness durations were similar and strong circadian rhythmicity was evident.

### Effects of lighting on behaviour measures

The lack of a treatment effect on spontaneous blink rates could be attributed to the management conditions of the studied group, which provided visual contact with conspecifics, ample forage, daily exercise and sufficient environmental stimulation with regular human interaction. In this low-stress context, the stress coping benefits afforded by the provision of optimised light spectra and intensities may not have been sufficient to induce blink rate changes within the studied population. In support of this notion, horse based studies that utilised SBR as a stress indicator applied clearly defined acute stressors such as sham clipping [48] and sudden arrival of a novel object [47]. As such, future studies featuring higher stress management conditions are required to explore further the relationship between lighting conditions and stress.

The horses in the study achieved an average total sleep time of three hours, but with substantial variation between individuals. These results align with findings of previous studies in stabled horses where a range of total sleep time from 2.5 to 5 h was reported [8]. No difference in total sleep time was observed between treatment and control groups, but as expected, sleep duration significantly differed between lighting periods (Day and Night). In agreement a with previous study, it was observed that horses carried out most sleep behaviour during the night [55]. As diurnally active prey animals, horses may feel less safe and exhibit greater vigilance during daylight hours when near people and other external stimuli [56]. Greater wakefulness exhibited by day was therefore expected, but significant differences in wakefulness duration between day and night were only observed under the treatment condition. One explanation for this relates to the known increased suppressive effect of blue-enriched lighting on melatonin production [35]. This is supported by the substantially lower levels of blue wavelengths in the spectral composition of the fluorescent lights used in the control condition, and the higher daytime light intensities recorded at eye level under the treatment LED lighting. Blue light is known for enhancing wakefulness in humans [57–60] and is also known to improve alertness in the workplace [61]. Our results suggest that the differences in proportion of blue light emitted by the two lighting conditions may have influenced alertness during daytime.

Horses were observed to spend more time standing during the day than at night. This finding is not unexpected as horses tend to exhibit more sleeping behaviour during the night [55]. It is common among horses to spend significant time standing during the day [62]. In feral horses, standing behaviour was described as a resting state characterised by a sense of ease and relaxation [63]. Standing can be seen as a resting behaviour as horses adopt the stay apparatus position as they stand at rest [64], allowing them to do so with minimal muscular effort. In this study, observers employed to study video files were found to significantly agree in their scores, however further clarification is required to help differentiate between standing awake, standing at rest (drowsiness) and standing sleep to increase the accuracy of reporting within the equine sleep literature [65]. While a distinct head drop is often indicative of the transition between drowsiness and sleep while standing [64], this was not always clearly perceived in all horses, requiring increased reliance on observation of partially or fully closed eyes and reduced blinks and ear movements to indicate sleep.

Unusually, Horse 1 did not display any recumbent behaviour during the six days of video observation contributing to a low total sleep duration. Although we do not know if the horse displayed recumbency behaviour on non-recorded nights of the study, it could be a welfare concern as lack of recumbency suggests REM sleep deprivation [66]. However, frequent daily horse welfare checks did not note any abnormalities for this animal. The observer also reported that this horse displayed frequent atypical behaviour at night, involving short periods during standing where the horse became unsteady, its head dropped, and its muzzle touched the ground. Such behaviour has been described for horses that do not lie down [64] and requires further investigation. Important to note was that the mean duration of ‘Unknown’ behaviour for Horse 1, where his head was over the stable door, was considerably longer at 6.5 h, compared to the mean ‘Unknown’ duration for all horses of 1.8 h. This difference implies that a significant proportion of sleep time for this horse may have occurred while his head rested on the stable door.

The lack of difference in duration of sleep behaviours between treatment and control lighting conditions implies that dim red light at night does not impact the normal sleep patterns in horses compared to natural dark conditions. This is the first study to confirm that red light is a suitable alternative to darkness in stabled horses to maintain sleep behaviour. These results support a previous finding that the nighttime rise in melatonin was maintained under dim red light [39] and further support using red light at night to avoid the disruptive effects of white light on circadian rhythms [38].

### Effects of lighting on circadian oscillations in hair follicles

Hair follicles represent a peripheral clock in mammals that is synchronised by the master clock and entrained by the light-dark cycle [67]. The expression patterns of the clock genes, *PER2, NR1D2,* and the clock-controlled gene *DBP,* have been used previously to assess internal circadian synchrony in human and horse studies [23,24,36]. In this study, significant circadian oscillations of *PER2* and *DBP* were observed under both lighting conditions indicating that both provided sufficient entrainment cues to the circadian system. Unexpectedly, *NR1D2* did not vary over time in contrast to previous findings. The lack of rhythmicity for this clock gene transcript may be due to the different stage of the hair growth cycle of the study horses as a result of exposure to a different photoperiod length compared to previous studies. In Collery et al., (2023), for example, horses were exposed to a long day photoperiod consisting of 17 h of light, contrasting with the photoperiod length of 13 h of light in the current study [36]. The duration of melatonin secretion, which mirrors the hours of darkness within the photoperiod, regulates the circannual pattern of prolactin secretion, and in turn the hair growth cycle [68–70]. It is postulated therefore that the lack of rhythmicity in NR1D2 may relate to the reported different roles played by specific clock genes in the regulation of the hair cycle at different growth stages in response to differing photoperiods [71].

Previously, stabled racehorses maintained under control conditions consisting of erratic nighttime exposure to incandescent light did not demonstrate rhythmic clock gene expression in hair follicles [36]. However, when switched to a customised LED lighting system, similar to that used in the current study, significant circadian clock gene rhythmicity for *NR1D2* and *PER2* was reported [36]. The authors concluded that the treatment lighting provided a robust entrainment signal to the circadian system, noting that red light at night enabled handlers to perform their duties during hours of darkness while avoiding the suppressive effects on melatonin production that switching on of white lights is known to induce. In contrast, the exposure to uninterrupted darkness experienced under the control lighting condition in the current study, which is known to facilitate the normal pattern of melatonin production, likely explains the differences in results in clock gene expression patterns between studies.

Many horse owners conserve energy by turning off barn lights during the day when not interacting with their horses. In modern equine husbandry, horses are often stabled for up to 23 h a day [72,73] and in the current study horses were stabled for at least 22 h. However, the experimental conditions in the current study were such that all horses experienced uninterrupted exposure to artificial light from morning until evening. This consistent exposure to daytime lighting likely contributed to the observed rhythmic clock gene expression patterns in all study horses. Further exploration of circadian rhythm and behaviour patterns of horses exposed to lighting programmes that vary in consistency and spectral composition would permit the development of best practice guidelines for stabled horses.

### Limiting factors

Due to the objectives of this study, such that the impacts of lighting were assessed in horses housed in their usual stables on a working yard, it was not feasible to block natural light from barn doorways. Daylight is naturally high in blue light, and due to the positioning of the stables within the barn, it is likely that some of this reached the study horses during the trial periods, especially when they hung their heads over the stable doors. This may have compromised the control lighting condition to some extent by increasing the blue light exposure during the day. More insights may be gained in future controlled studies where stables lack exposure to natural light.

Finally, the large inter-individual differences observed between horses in relation to their sleep behaviour patterns, especially Horse 1, may have limited the ability to detect differences in some cases, despite the implementation of a cross-over study design. Pre-screening of study animals to select for horses that clearly exhibit sleep durations within a range considered normal for the species may represent a valid consideration for future similar studies.

## Conclusion

No overall significant differences in arousal, sleep behaviour or clock gene rhythmicity in a peripheral tissue were observed in horses exposed to fluorescent daytime light and darkness at night or a customised LED lighting system comprising blue-enriched daytime light and red light at night. The horses in this study were well managed regarding adherence to turning lights on and off at regular times in the morning and evening. Therefore, the lack of significant findings is not unexpected given the lack of nighttime light disturbances under the control lighting condition. The findings also suggest that dim red light at night is a suitable light spectrum to facilitate normal sleep behaviour in horses as well as maintaining internal circadian synchrony. These results contribute to our understanding of how stable lighting can promote healthy sleep behaviour patterns and optimise circadian rhythms in horses, providing a foundation to inform future research in this important area.

## Supporting information

S1 Table Equine candidate clock gene and internal reference gene sequences used for qPCR. (DOCX)

## References

[1] Williams J, Tabor G. Rider impacts on equitation. Appl Anim Behav Sci. 2017;190:28–42.

[2] McGreevy PD, McLean A. Behavioural problems with the ridden horse. In: Mills M, McDonnell S, editors. The domestic horse: The origins, development and management of its behaviour. Cambridge: Cambridge University Press; 2005. p. 196–211.

[3] BETA. British Equestrian Trade Association National Equestrian Survey: Structural Report; 2023 [cited 12 May 2025] Available from: https://beta-uk.org/industry-information/

[4] Lesimple C, Poissonnet A, Hausberger M. How to keep your horse safe? An epidemiological study about management practices. Appl Anim Behav Sci. 2016;181:105–14.

[5] Greening L, Downing J, Amiouny D, Lekang L, McBride S. The effect of altering routine husbandry factors on sleep duration and memory consolidation in the horse. Appl Anim Behav Sci. 2021;236:105229.

[6] Greening L, Allen S, McBride S. Towards an objective measurement of sleep quality in non-human animals: using the horse as a model species for the creation of sleep quality indices. Biol Open. 2023;12(7):bio059964. doi: 10.1242/bio.059964 37378461 PMC10373578

[7] Deboer T. Sleep homeostasis and the circadian clock: Do the circadian pacemaker and the sleep homeostat influence each other’s functioning? Neurobiol Sleep Circadian Rhythms. 2018;5:68–77. doi: 10.1016/j.nbscr.2018.02.003 31236513 PMC6584681

[8] Greening L, McBride S. A review of equine sleep: Implications for equine welfare. Front Vet Sci. 2022;9:916737. 36061116 10.3389/fvets.2022.916737 PMC9428463

[9] Prayag AS, Münch M, Aeschbach D, Chellappa SL, Gronfier C. Light modulation of human clocks, wake, and sleep. Clocks Sleep. 2019;1(1):193–208. doi: 10.3390/clockssleep1010017 32342043 PMC7185269

[10] Dijk D-J, Archer SN. Light, sleep, and circadian rhythms: together again. PLoS Biol. 2009;7(6):e1000145. doi: 10.1371/journal.pbio.1000145 19547745 PMC2691600

[11] Langford FM, Cockram MS. Is sleep in animals affected by prior waking experiences? Anim Welf. 2010;19(3):215–22.

[12] Greening L, Pullen A, Tame S, Bufton CA. The welfare implications of horse owner perceptions and knowledge of equine sleep. In: Greening L, editor. Virtual UFAW Animal Welfare Conference [Internet]. 2020. Available from: https://pure.hartpury.ac.uk/en/publications/the-welfare-implications-of-owner-perceptions-and-knowledge-of-eq

[13] Kuhlman SJ, Mackey SR, Duffy JF. Biological Rhythms Workshop I: Introduction to Chronobiology. In: Cold Spring Harbor Symposia on Quantitative Biology, vol. 72. Cold Spring Harbor Laboratory Press; 2007. p. 1–6. 10.1101/sqb.2007.72.059 18419258

[14] Lucas RJ, Freedman MS, Lupi D, Munoz M, David-Gray ZK, Foster RG. Identifying the photoreceptive inputs to the mammalian circadian system using transgenic and retinally degenerate mice. Behav Brain Res. 2001;125(1–2):97–102. doi: 10.1016/s0166-4328(01)00274-1 11682100

[15] Moore RY. Organization and function of a central nervous system circadian oscillator: the suprachiasmatic hypothalamic nucleus. Fed Proc. 1983;42(11):2783–9. 6135628

[16] Ralph MR, Foster RG, Davis FC, Menaker M. Transplanted suprachiasmatic nucleus determines circadian period. Science. 1990;247(4945):975–8. doi: 10.1126/science.2305266 2305266

[17] Guo H, Brewer JM, Champhekar A, Harris RBS, Bittman EL. Differential control of peripheral circadian rhythms by suprachiasmatic-dependent neural signals. Proc Natl Acad Sci U S A. 2005;102(8):3111–6. doi: 10.1073/pnas.0409734102 15710878 PMC548796

[18] Pezük P, Mohawk JA, Wang LA, Menaker M. Glucocorticoids as entraining signals for peripheral circadian oscillators. Endocrinology. 2012;153(10):4775–83. doi: 10.1210/en.2012-1486 22893723 PMC3512018

[19] Murphy BA. Circadian and Circannual Regulation in the Horse: Internal Timing in an Elite Athlete. J Equine Vet Sci. 2019;76:14–24. doi: 10.1016/j.jevs.2019.02.026 31084748

[20] Stephan FK, Zucker I. Circadian rhythms in drinking behavior and locomotor activity of rats are eliminated by hypothalamic lesions. Proc Natl Acad Sci U S A. 1972;69(6):1583–6. doi: 10.1073/pnas.69.6.1583 4556464 PMC426753

[21] Tahara Y, Kuroda H, Saito K, Nakajima Y, Kubo Y, Ohnishi N, et al. In vivo monitoring of peripheral circadian clocks in the mouse. Curr Biol. 2012;22(11):1029–34. doi: 10.1016/j.cub.2012.04.009 22578421

[22] Yoo S-H, Yamazaki S, Lowrey PL, Shimomura K, Ko CH, Buhr ED, et al. PERIOD2::LUCIFERASE real-time reporting of circadian dynamics reveals persistent circadian oscillations in mouse peripheral tissues. Proc Natl Acad Sci U S A. 2004;101(15):5339–46. doi: 10.1073/pnas.0308709101 14963227 PMC397382

[23] Watts LM, Browne JA, Murphy BA. Investigation of a non-invasive method of assessing the equine circadian clock using hair follicle cells. J Circadian Rhythms. 2012;10(1):7. doi: 10.1186/1740-3391-10-7 23039139 PMC3514281

[24] Akashi M, Soma H, Yamamoto T, Tsugitomi A, Yamashita S, Yamamoto T, et al. Noninvasive method for assessing the human circadian clock using hair follicle cells. Proc Natl Acad Sci U S A. 2010;107(35):15643–8. doi: 10.1073/pnas.1003878107 20798039 PMC2932591

[25] Pevet P, Challet E, Felder-Schmittbuhl M-P. Melatonin and the circadian system: Keys for health with a focus on sleep. Handb Clin Neurol. 2021;179:331–43. doi: 10.1016/B978-0-12-819975-6.00021-2 34225973

[26] Moore RY, Lenn NJ. A retinohypothalamic projection in the rat. J Comp Neurol. 1972;146(1):1–14. doi: 10.1002/cne.901460102 4116104

[27] Bartness TJ, Goldman BD. Mammalian pineal melatonin: a clock for all seasons. Experientia. 1989;45(10):939–45. doi: 10.1007/BF01953051 2680574

[28] Paredes SD, Terrón MP, Valero V, Barriga C, Reiter RJ, Rodríguez AB. Orally administered melatonin improves nocturnal rest in young and old ringdoves (Streptopelia risoria). Basic Clin Pharmacol Toxicol. 2007;100(4):258–68. doi: 10.1111/j.1742-7843.2006.00032.x 17371530

[29] Markus RP, Ferreira ZS, Fernandes PACM, Cecon E. The immune-pineal axis: a shuttle between endocrine and paracrine melatonin sources. Neuroimmunomodulation. 2007;14(3–4):126–33. doi: 10.1159/000110635 18073503

[30] Touitou Y, Reinberg A, Touitou D. Association between light at night, melatonin secretion, sleep deprivation, and the internal clock: Health impacts and mechanisms of circadian disruption. Life Sci. 2017;173:94–106. doi: 10.1016/j.lfs.2017.02.008 28214594

[31] Brainard GC, Sliney D, Hanifin JP, Glickman G, Byrne B, Greeson JM, et al. Sensitivity of the human circadian system to short-wavelength (420-nm) light. J Biol Rhythms. 2008;23(5):379–86. doi: 10.1177/0748730408323089 18838601

[32] Gillette M. Introduction to biological timing in health and disease. In: Gillette M, editor. Chronobiology: Biological timing in health and disease. Academic Press; 2013. p. xi–xvi. 10.1016/B978-0-12-396971-2.10000-4 23899603

[33] Bonmati-Carrion MA, Arguelles-Prieto R, Martinez-Madrid MJ, Reiter R, Hardeland R, Rol MA, et al. Protecting the melatonin rhythm through circadian healthy light exposure. Int J Mol Sci. 2014;15(12):23448–500. doi: 10.3390/ijms151223448 25526564 PMC4284776

[34] Foster RG, Hankins MW, Peirson SN. Light, Photoreceptors, and Circadian Clocks. In: Rosato E, editor. Circadian Rhythms. Methods in Molecular Biology™. Humana Press; 2007. doi: 10.1007/978-1-59745-257-1_1 17416998

[35] Walsh CM, Prendergast RL, Sheridan JT, Murphy BA. Blue light from light-emitting diodes directed at a single eye elicits a dose-dependent suppression of melatonin in horses. Vet J. 2013;196(2):231–5. doi: 10.1016/j.tvjl.2012.09.003 23079244

[36] Collery A, Browne JA, O’Brien C, Sheridan JT, Murphy BA. Optimised Stable Lighting Strengthens Circadian Clock Gene Rhythmicity in Equine Hair Follicles. Animals (Basel). 2023;13(14):2335. doi: 10.3390/ani13142335 37508112 PMC10376498

[37] Redlin U. Neural basis and biological function of masking by light in mammals: suppression of melatonin and locomotor activity. Chronobiol Int. 2001;18(5):737–58. doi: 10.1081/cbi-100107511 11763983

[38] Murphy BA, Elliott JA, Sessions DR, Vick MM, Kennedy EL, Fitzgerald BP. Rapid phase adjustment of melatonin and core body temperature rhythms following a 6-h advance of the light/dark cycle in the horse. J Circadian Rhythms. 2007;5:5. doi: 10.1186/1740-3391-5-5 17718919 PMC2020455

[39] Murphy BA, O’Brien C, Elliott JA. Red light at night permits the nocturnal rise of melatonin production in horses. Vet J. 2019;252:105360. doi: 10.1016/j.tvjl.2019.105360 31554596

[40] Morita T, Tokura H. The influence of different wavelengths of light on human biological rhythms. Appl Human Sci. 1998;17(3):91–6. doi: 10.2114/jpa.17.91 9682518

[41] Figueiro MG, Rea MS. The effects of red and blue lights on circadian variations in cortisol, alpha amylase, and melatonin. Int J Endocrinol. 2010;2010:829351. doi: 10.1155/2010/829351 20652045 PMC2905913

[42] Stenvers DJ, van Dorp R, Foppen E, Mendoza J, Opperhuizen A-L, Fliers E, et al. Dim light at night disturbs the daily sleep-wake cycle in the rat. Sci Rep. 2016;6:35662. doi: 10.1038/srep35662 27762290 PMC5071835

[43] Opperhuizen A-L, Stenvers DJ, Jansen RD, Foppen E, Fliers E, Kalsbeek A. Light at night acutely impairs glucose tolerance in a time-, intensity- and wavelength-dependent manner in rats. Diabetologia. 2017;60(7):1333–43. doi: 10.1007/s00125-017-4262-y 28374068 PMC5487588

[44] Zhang Z, Wang H-J, Wang D-R, Qu W-M, Huang Z-L. Red light at intensities above 10 lx alters sleep-wake behavior in mice. Light Sci Appl. 2017;6(5):e16231. doi: 10.1038/lsa.2016.231 30167247 PMC6062196

[45] Hirotsu C, Tufik S, Andersen ML. Interactions between sleep, stress, and metabolism: From physiological to pathological conditions. Sleep Sci. 2015;8(3):143–52. doi: 10.1016/j.slsci.2015.09.002 26779321 PMC4688585

[46] McBride SD, Roberts K, Hemmings AJ, Ninomiya S, Parker MO. The impulsive horse: Comparing genetic, physiological and behavioral indicators to those of human addiction. Physiol Behav. 2022;254:113896. doi: 10.1016/j.physbeh.2022.113896 35777460

[47] Merkies K, Ready C, Farkas L, Hodder A. Eye Blink Rates and Eyelid Twitches as a Non-Invasive Measure of Stress in the Domestic Horse. Animals (Basel). 2019;9(8):562. doi: 10.3390/ani9080562 31443315 PMC6721043

[48] Mott RO, Hawthorne SJ, McBride SD. Blink rate as a measure of stress and attention in the domestic horse (Equus caballus). Sci Rep. 2020;10(1):21409. doi: 10.1038/s41598-020-78386-z 33293559 PMC7722727

[49] Equilume. Equilume performance lighting: Stable light product manual. 2024. Available from: https://equilume.com/aus/wp-content/uploads/sites/3/2024/08/Stable-Light-Product-Manual-2024V1-07.2024.pdf

[50] Evans L, Cameron-Whytock H, Ijichi C. Eye understand: Physiological measures as novel predictors of adaptive learning in horses. Appl Anim Behav Sci. 2024;271:106152.

[51] Lelláková M, Pavľak A, Florián M, Lešková L, Takáčová D, Kottferová J. Monitoring of Stress in Police Horses. Folia Veterinaria. 2021;65(1):54–8. doi: 10.2478/fv-2021-0007

[52] Harkin EE, Browne JA, Murphy BA. Evaluation of short-term hair follicle storage conditions for maintenance of RNA integrity. PLoS One. 2024;19(5):e0294089. doi: 10.1371/journal.pone.0294089 38820307 PMC11142484

[53] Refinetti R, Cornélissen G, Halberg F. Procedures for numerical analysis of circadian rhythms. Biol Rhythm Res. 2007;38(4):275–325. 23710111 10.1080/09291010600903692 PMC3663600

[54] Halberg F, Tong YL, Johnson EA. Circadian system phase—an aspect of temporal morphology; procedures and illustrative examples. In: von Mayersbach H, editor. The Cellular Aspects of Biorhythms: Symposium on Rhythmic Research Sponsored by the VIIIth International Congress of Anatomy Wiesbaden. Berlin Heidelberg: Springer; 1967. p. 20–48.

[55] Stachurska A, Kowalska N, Kolstrung R, Pluta M. Time of lying in the horse with regard to its sex and system of management. Acta Sci Pol Zootech. 2016;15(2):77–86. doi: 10.21005/asp.2016.15.2.07

[56] McNamara JM, Barta Z, Fromhage L, Houston AI. The coevolution of choosiness and cooperation. Nature. 2008;451(7175):189–92. doi: 10.1038/nature06455 18185587

[57] Phipps-Nelson J, Redman JR, Schlangen LJ, Rajaratnam SM. Blue light exposure reduces objective measures of sleepiness during prolonged nighttime performance testing. Chronobiol Int. 2009;26(5):891–912. 19637049 10.1080/07420520903044364

[58] Lockley SW, Gooley JJ. Circadian photoreception: spotlight on the brain. Curr Biol. 2006;16(18):R795-7. doi: 10.1016/j.cub.2006.08.039 16979545

[59] Alkozei A, Smith R, Pisner DA, Vanuk JR, Berryhill SM, Fridman A, et al. Exposure to blue light increases subsequent functional activation of the prefrontal cortex during performance of a working memory task. Sleep. 2016;39(9):1671–80. doi: 10.5665/sleep.6090 27253770 PMC4989256

[60] Choi K, Shin C, Kim T, Chung HJ, Suk H-J. Awakening effects of blue-enriched morning light exposure on university students’ physiological and subjective responses. Sci Rep. 2019;9(1):345. doi: 10.1038/s41598-018-36791-5 30674951 PMC6344573

[61] Viola AU, James LM, Schlangen LJM, Dijk D-J. Blue-enriched white light in the workplace improves self-reported alertness, performance and sleep quality. Scand J Work Environ Health. 2008;34(4):297–306. doi: 10.5271/sjweh.1268 18815716

[62] Gellman K, Ruina A. Standing horse posture: a longer stance is more stable. Biol Open. 2022;11(4):bio059139. doi: 10.1242/bio.059139 35545924 PMC9115912

[63] Ransom JI, Cade BS. Quantifying equid behavior—a research ethogram for free-roaming feral horses. US Geol Surv Tech Methods. 2009;26:2-A9.

[64] Williams DC, Aleman M, Holliday TA, Fletcher DJ, Tharp B, Kass PH, et al. Qualitative and quantitative characteristics of the electroencephalogram in normal horses during spontaneous drowsiness and sleep. J Vet Intern Med. 2008;22(3):630–8. doi: 10.1111/j.1939-1676.2008.0096.x 18466241

[65] Borthwick EJ, Preshaw L, Wheeler-Launder C, Challinor C, Housby-Skeggs N, Boalch E, et al. Stable design influences relaxation and affiliative behavior in horses during short isolation bouts. J Vet Behav. 2023;69:1–7.

[66] Aleman M, Williams C, Holliday T. Sleep and sleep disorders in horses. Proc Annu Meet Am Assoc Equine Pract. 2008;54:180–5.

[67] Liu L-P, Li M-H, Zheng Y-W. Hair Follicles as a Critical Model for Monitoring the Circadian Clock. Int J Mol Sci. 2023;24(3):2407. doi: 10.3390/ijms24032407 36768730 PMC9916850

[68] Fitzgerald BP, Davison LA, McManus CJ. Evidence for a seasonal variation in the ability of exogenous melatonin to suppress prolactin secretion in the mare. Domest Anim Endocrinol. 2000;18(4):395–408. doi: 10.1016/s0739-7240(00)00058-8 10869858

[69] Foitzik K, Krause K, Nixon AJ, Ford CA, Ohnemus U, Pearson AJ, et al. Prolactin and its receptor are expressed in murine hair follicle epithelium, show hair cycle-dependent expression, and induce catagen. Am J Pathol. 2003;162(5):1611–21. doi: 10.1016/S0002-9440(10)64295-2 12707045 PMC1851183

[70] O’Brien C, Darcy-Dunne MR, Murphy BA. The effects of extended photoperiod and warmth on hair growth in ponies and horses at different times of year. PLoS One. 2020;15(1):e0227115. doi: 10.1371/journal.pone.0227115 31935219 PMC6959597

[71] Niu Y, Wang Y, Chen H, Liu X, Liu J. Overview of the Circadian Clock in the Hair Follicle Cycle. Biomolecules. 2023;13(7):1068. doi: 10.3390/biom13071068 37509104 PMC10377266

[72] Wylie CE, Ireland JL, Collins SN, Verheyen KLP, Newton JR. Demographics and management practices of horses and ponies in Great Britain: a cross-sectional study. Res Vet Sci. 2013;95(2):410–7. doi: 10.1016/j.rvsc.2013.05.004 23735292

[73] Hockenhull J, Furtado T. Escaping the gilded cage: could COVID-19 lead to improved equine welfare? A review of the literature. Appl Anim Behav Sci. 2021;237:105303.

