# Supplementary material for: Influence of lighting on sleep behaviour, circadian rhythm and spontaneous blink rate in stabled riding school horses (Equus caballus)
Source: PLoS One. 2025 Jun 27;20(6):e0326567. doi: 10.1371/journal.pone.0326567 (PMC12204570; doi:10.1371/journal.pone.0326567)
Supplement: S1 Table — (DOCX) [file pone.0326567.s001.docx]

S1 Table. Equine candidate clock gene and internal reference gene sequences used for qPCR

| **Gene Symbol** | **Forward 5’ – 3’** | **Reverse 5’ – 3’** |
| --- | --- | --- |
| *PER2* | AGCCTGATGATGGCGAAGTCTGAA | AGTTCTTTGTGCGTGTCTGCCTTG |
| *NR1D2* | CAACACTGGAGGGAGAATGC | CTGGGGTAAAGCTCATCGAA |
| *RPL19* | CTGATCATCCGGAAGCCTGT | GGCAGTACCCTTTCGCTTAC |
| *PPIA* | GCATCTTGTCCATGGCGAAT | CAAAGACCACATGCTTGCCA |
| *H3F3A* | CAAACTTCCCTTCCAGCGTC | TGGATAGCACACAGGTTGGT |
| *RNF11* | TGTTGTGTCTCGGTCCATGA | AGATCATGGCCGTCTCTGAA |
| *YWHAZ* | AGACGGAAGGTGCTGAGAAA | CTTGTGAAGCATTGGGGATCA |
| *SDHA* | AGGATAGCTCAAAGAATAGGCCT | CGGCAGAAATCGAATTGGGT |
| *GADPH* | GGAGTCCACTGGTGTCTTCA | GTTCACGCCCATCACAAACA |
| *ACTB* | CACCTTCTACAACGAGCTGC | CGGGGTGTTGAAGGTCTCA |
