# Supplementary material for: Influence of lighting on sleep behaviour, circadian rhythm and spontaneous blink rate in stabled riding school horses (Equus caballus)
Source: PLoS One. 2025 Jun 27;20(6):e0326567. doi: 10.1371/journal.pone.0326567 (PMC12204570; doi:10.1371/journal.pone.0326567)
Supplement: Attachment — Submitted filename: Response to Reviewers.docx [file pone.0326567.s002.docx]

Dear Editor and Reviewers,

We are very grateful for the detailed and thoughtful comments by both reviewers. We have amended our manuscript extensively in light of comments and provide a point-by-point rebuttal (in black font) below. The amendments have significantly improved the clarity, impact and conciseness of the manuscript which we hope you will find is now suitable for publication.

Barbara Murphy

PONE-D-24-58876

Influence of lighting on sleep behaviour, circadian rhythm and spontaneous blink rate in stabled riding school horses (Equus caballus)

PLOS ONE

Dear Dr. Murphy,

Thank you for submitting your manuscript to PLOS ONE. After careful consideration, we feel that it has merit but does not fully meet PLOS ONE’s publication criteria as it currently stands. Therefore, we invite you to submit a revised version of the manuscript that addresses the points raised during the review process.

The requested changes are both in style and in substance. Specifically, the manuscript is both verbose and repetitive. The manuscript is long and could be significantly shortened without diminishing its impact by correcting these issues and by removing some items, as suggested by the reviewers.

Thank you. We agree and have now shortened the manuscript significantly by removing some sections entirely, shortening others and condensing Figures.

Furthermore, the link between SBR and sleep deprivation, as it pertains to horses, is circumstantial and the authours should be cautious with the interpretations of these data.

Thank you. In response to the editor’s comment, and comments from Reviewer 2, we have shifted the focus of the general narrative concerning blink rate, and the discussion of the results, away from blink rate as a measure of dopamine and sleep deprivation, towards what blink rate is telling us about the animal’s stress response.

Please submit your revised manuscript by Apr 12 2025 11:59PM. If you will need more time than this to complete your revisions, please reply to this message or contact the journal office at [plosone@plos.org](mailto:plosone@plos.org). When you're ready to submit your revision, log on to <https://www.editorialmanager.com/pone/> and select the 'Submissions Needing Revision' folder to locate your manuscript file.

Please include the following items when submitting your revised manuscript:

- A rebuttal letter that responds to each point raised by the academic editor and reviewer(s). You should upload this letter as a separate file labeled 'Response to Reviewers'.
- A marked-up copy of your manuscript that highlights changes made to the original version. You should upload this as a separate file labeled 'Revised Manuscript with Track Changes'.
- An unmarked version of your revised paper without tracked changes. You should upload this as a separate file labeled 'Manuscript'.

If you would like to make changes to your financial disclosure, please include your updated statement in your cover letter. Guidelines for resubmitting your figure files are available below the reviewer comments at the end of this letter.

If applicable, we recommend that you deposit your laboratory protocols in [protocols.io](http://protocols.io/) to enhance the reproducibility of your results. Protocols.io assigns your protocol its own identifier (DOI) so that it can be cited independently in the future. For instructions see: [https://journals.plos.org/plosone/s/submission-guidelines#loc-laboratory-protocols](https://track.editorialmanager.com/CL0/https:%2F%2Fjournals.plos.org%2Fplosone%2Fs%2Fsubmission-guidelines%23loc-laboratory-protocols/1/010f019543540d38-b37e0b64-93a7-4be1-b277-9b296f535f26-000000/CM-0U0sk8IzxPJDWarwlMJtyvdC9SDY4s3mQTTSpM1s=199). Additionally, PLOS ONE offers an option for publishing peer-reviewed Lab Protocol articles, which describe protocols hosted on [protocols.io](http://protocols.io/). Read more information on sharing protocols at [https://plos.org/protocols?utm_medium=editorial-email&utm_source=authorletters&utm_campaign=protocols](https://track.editorialmanager.com/CL0/https:%2F%2Fplos.org%2Fprotocols%3Futm_medium=editorial-email%26utm_source=authorletters%26utm_campaign=protocols/1/010f019543540d38-b37e0b64-93a7-4be1-b277-9b296f535f26-000000/RNTVlWPL5WVr_By9fBw53elEkJHBU4gUq9czp5lR8UI=199).

We look forward to receiving your revised manuscript.

Kind regards,

Paul A. Bartell
Academic Editor
PLOS ONE

Journal requirements:

When submitting your revision, we need you to address these additional requirements.

1. Please ensure that your manuscript meets PLOS ONE's style requirements, including those for file naming. The PLOS ONE style templates can be found at

<https://journals.plos.org/plosone/s/file?id=wjVg/PLOSOne_formatting_sample_main_body.pdf> and

Thank you, we have followed formatting guidance to address the issues identified namely:

- Changed format of headings
- Amended in text citation to include hyphens not commas where there were multiple sources
- Changed figure to fig/figs and amended titles/legends for tables
- Added page numbers
- Corrected reference formatting in reference list

[https://journals.plos.org/plosone/s/file?id=ba62/PLOSOne_formatting_sample_title_authors_affiliations.pdf](https://track.editorialmanager.com/CL0/https:%2F%2Fjournals.plos.org%2Fplosone%2Fs%2Ffile%3Fid=ba62%2FPLOSOne_formatting_sample_title_authors_affiliations.pdf/1/010f019543540d38-b37e0b64-93a7-4be1-b277-9b296f535f26-000000/ULL00q920HO4cb-IAfUrjCe8hbfTf5S543dQYVdk7ps=199).

2. Please amend your list of authors on the manuscript to ensure that each author is linked to an affiliation. Authors’ affiliations should reflect the institution where the work was done (if authors moved subsequently, you can also list the new affiliation stating “current affiliation:….” as necessary).

We are confident that all authors are linked to an affiliation and updated the current address for one.

3. Please provide captions for Figure 9 in your manuscript.

This caption is provided in the manuscript.

4.  Thank you for stating the following in the Competing Interests section:

[I have read the journal's policy and the authors of this manuscript have the following competing interests: BAM is the Founder of Equilume Ltd., a spin-out company deriving from her research program as associate professor at University College Dublin and is a member of the company’s Board of Directors. BAM is a shareholder in Equilume Ltd. PK is an employee of Equilume Ltd. The treatment lighting condition in the present study comprised an Equilume Stable Light and is a commercially available product.].

Please confirm that this does not alter your adherence to all PLOS ONE policies on sharing data and materials, by including the following statement: ""This does not alter our adherence to  PLOS ONE policies on sharing data and materials.” (as detailed online in our guide for authors <http://journals.plos.org/plosone/s/competing-interests>).  If there are restrictions on sharing of data and/or materials, please state these. Please note that we cannot proceed with consideration of your article until this information has been declared.

Please include your updated Competing Interests statement in your cover letter; we will change the online submission form on your behalf.

The updated Competing Interest Statement has now been included in our cover letter.

5. Please provide a complete Data Availability Statement in the submission form, ensuring you include all necessary access information or a reason for why you are unable to make your data freely accessible. If your research concerns only data provided within your submission, please write "All data are in the manuscript and/or supporting information files" as your Data Availability Statement.

6. Please include captions for your Supporting Information files at the end of your manuscript, and update any in-text citations to match accordingly. Please see our Supporting Information guidelines for more information: <http://journals.plos.org/plosone/s/supporting-information>.

This has been done.

[Note: HTML markup is below. Please do not edit.]

Reviewers' comments:

Reviewer's Responses to Questions

**Comments to the Author**

1. Is the manuscript technically sound, and do the data support the conclusions?

The manuscript must describe a technically sound piece of scientific research with data that supports the conclusions. Experiments must have been conducted rigorously, with appropriate controls, replication, and sample sizes. The conclusions must be drawn appropriately based on the data presented.

Reviewer #1: Yes

Reviewer #2: Partly

2. Has the statistical analysis been performed appropriately and rigorously?

Reviewer #1: Yes

Reviewer #2: I Don't Know

3. Have the authors made all data underlying the findings in their manuscript fully available?

The [PLOS Data policy](http://track.editorialmanager.com/CL0/http:%2F%2Fwww.plosone.org%2Fstatic%2Fpolicies.action%23sharing/1/010f019543540d38-b37e0b64-93a7-4be1-b277-9b296f535f26-000000/n3j58Jf0D5Dj-VhRG-7rNr1l43SurZvxy-UjcY06lzs=199) requires authors to make all data underlying the findings described in their manuscript fully available without restriction, with rare exception (please refer to the Data Availability Statement in the manuscript PDF file). The data should be provided as part of the manuscript or its supporting information, or deposited to a public repository. For example, in addition to summary statistics, the data points behind means, medians and variance measures should be available. If there are restrictions on publicly sharing data—e.g. participant privacy or use of data from a third party—those must be specified.

Reviewer #1: Yes

Reviewer #2: Yes

4. Is the manuscript presented in an intelligible fashion and written in standard English?

PLOS ONE does not copyedit accepted manuscripts, so the language in submitted articles must be clear, correct, and unambiguous. Any typographical or grammatical errors should be corrected at revision, so please note any specific errors here.

Reviewer #1: Yes

Reviewer #2: Yes

5. Review Comments to the Author

Please use the space provided to explain your answers to the questions above. You may also include additional comments for the author, including concerns about dual publication, research ethics, or publication ethics. (Please upload your review as an attachment if it exceeds 20,000 characters)

Reviewer #1: Overall comments: An interesting study. I enjoyed reading this manuscript. There are some edits to be made, and those are noted below.

Specific comments:

Line 50-51: please recheck grammar for this sentence, it seems choppy jumping from talking about environmental factors in horses and then light stimuli. Perhaps it needs to be 2 separate sentences?

Thank you. This sentence has been rechecked and improved for clarity.

Line 57-60: need an additional comma after hypothalamus

This edit has been made.

Line 76-77: tie back in the connect to melatonin in the final sentence of this paragraph.

Thank you. We have tied this point back in as follows: “Therefore, consistent sleep-wake cycles and healthy functioning of the circadian system requires the absence of light at night *to facilitate the normal rise in melatonin production* [30]”.

Line 78-81: what about melatonin? The light type that stimulates the circadian clock is discussed, but the way the sentence structure is set up, it feels like it is currently lacking a mention of the optimal stimulation for melatonin. OR – rephrase the sentence. Perhaps the part after the semicolon should just be its own sentence.

Thank you. We have made the section after the semicolon its own sentence, improving the clarity. Melatonin regulation by light is then addressed further on in the paragraph.

Line 97-101: is there research in any other mammalian species on red light and its impact on melatonin and the circadian rhythm?

There is in rodents, white light intensities as low as 5 lux have been shown to disrupt the sleep-wake cycle (Stenvers et al., 2016) whereas <10 lux of red light was shown to facilitate normal rest-activity behaviour and metabolic function (Zhang et al., 2017; Opperhuizen et al., 2017). We have now included these additional information and citations here.

Line 131-133: please provide number of mares and geldings, as well as breed.

The numbers of geldings and mares have been added. Specific breed details are not available other than to say they were mixed riding horse/cob breed types.

Line 142-143: what type of forage? Be more specific

The forage type has now been specified as hay.

Line 135: do you mean the stalls were partitioned by wooden panels? Not the stables?

Stalls (US) are termed ‘stables’ in the UK. In this line we state the size of individual stables within the larger barn that they are housed within.

Line 152: Table 1 – just to clarify – the treatments were two separate barns? Or the same barn? It is unclear from this picture. If it is two separate barns, I would label the table to clarify.

The treatments were in the same barn with both lighting treatments installed in each individual stable. This information is detailed beneath the table. This arrangement avoided any requirement to move horses to a different stable for the second lighting treatment, avoiding unnecessary disruption and the need for additional acclimatisation time. The caption has been amended as follows to improve clarity: “Schematic of the experimental barn layout and horse assignment to individual stables and groups (A and B) for cross-over design.”

Line 187 and line 188: barn and stable are used here interchangeably. Please pick one term to use and correct throughout paper.

Thank you. We have corrected this. ‘Stable’ is used for the individual horse enclosure and ‘barn’ used to describe the building the stables are housed within.

Line 194-195: So there was no washout period in between the two trials? Is there literature to show this has no effect on variables being measured?

There was no requirement for a wash-out period between the two lighting treatments as the control lighting represented the normal lighting regime that the horses were accustomed to. A 4-week experimental period was chosen as it can take two weeks for circadian rhythms to fully adapt to a new photoperiod in horses (Murphy *et al.*, 2007). However, even though there was little change in photoperiod between treatments for this study, all that changed was the intensity and spectral quality of the light, this buffer was allowed for and extended to ensure any and all behavioural parameters had fully adapted to the new lighting. The variables were then measured during week 4 of each treatment.

Line 200: I don’t think table 3 is really necessary. The protocol is explained sufficiently in the prior paragraph.

Thank you. We agree and have removed Table 3.

Line 347: consider moving the text in row 3 ( duration of behaviors in sec) to the bottom of the table – it’s a little jarring where it is now. And why for the results in this table are then H shown in all the rows, but not sec? I would either include both or neither, since you explain the units in row 3.

Respectfully, we feel it is best to define the units above the columns. However, we have shortened the description in row 3 so it looks less busy. We have also now removed the reference to hours (h) in all the rows as you correctly point out that these have previously been defined in row 3.

Line 438: was there any effect of age on the horses on behavior and arousal?

This effect was not examined as all horses bar one (aged 17) were aged from 7 to 15 years, classed as mature horses, not young (<7) or aged (>15). Plus, the groups were blocked for age to avoid any age-related confounding factors.

Line 489-498: Frankly, I do not this paragraph is necessary. There was no significant effect found by the researchers on time spent outside, and this did not seem to be a primary point of interest for the study. Especially considering the horses did different things when outside. While I agree looking at exercise and sleep patterns would be interesting, given the length of this paper, and sheer speculative manner of this particular paragraph, I would cut it.

Thank you. We agree and have now removed this entire paragraph from the manuscript.


Figure 2: are there better-quality images for this figure? The ones provided are a bit blurry.

The Figures uploaded on submission were high quality and did not appear blurry, but it is quite likely that they may only appear blurry on the generated pdf for review. We have checked that all Figures again again meet with journal specifications.

Figure 3: were any pre-trials done to see if the location of the go-pro impacted horses lying down to sleep? It seems based on this picture, if the go-pro happened to be on the side the horse preferred to be on when lying down, that this could skew results.

The horses were fitted with the go-pro attachment to record SBR for a 30-min duration only, during which time they were continuously observed while standing and did not lie down.

Figure 6-10: again, the resolution of these images seems a little blurry. Would recheck to make sure the best quality is uploaded for the manuscript.

Thank you. Each of these will be rechecked but we believe it is a result of the PDF generation

Reviewer #2: Review of “Influence of lighting on sleep behaviour, circadian rhythm and spontaneous blink rate in stabled riding school horses (Equus caballus)”.

Overview: This manuscript describes a study in which horses were exposed to 2 lighting sources and a host of behavioral and physiologic response variables were evaluated. The results indicate that in general there were not detectable differences between the treatment and control groups. There were clear differences in behavior between day and night and there was good evidence for circadian rhythms. This manuscript could be improved by focusing on the clear and obvious findings. It is likely that a manuscript that was 1/3 to ½ the length would be much more clear and concise and be of value to those that are conducting research in this area.

Strengths of this article include:
1. Focus on lighting and circadian patterns as they are associated with equine health and well-being. There is no doubt that this is an important area and in need of more work. The solution that is partially explored in this paper is feasible and makes good sense. There is data here that is one more piece of evidence in the growing literature in this area.
2. The use of well researched and developed LED lighting system is exciting and this manuscript is goo impetus to do more work in this area.

We thank the reviewer for highlighting some strengths of the manuscript.

Weaknesses of this article include:
1. The experimental design and potential negative impact of uncontrolled sources of variation make it nearly impossible to come to any clear conclusions.

Respectfully, the crossover experimental design employed in this experiment was specifically chosen due to its ability to significantly reduce sources of variation, the primary one being individual variation. This type of design eliminates or reduces the impact of individual variability, which can be a significant source of noise in between-subjects designs (like parallel group trials). As each horse acted as its own control and received both treatments, the power of the study increased significantly and equated to a similar study of > 20 horses without a crossover (parallel group trial). The design also accounted for a potential effect of time which creates additional potential for variation in a longitudinal study. Furthermore, this choice of design allowed for a reduced sample size, while increasing the precision of the estimated treatment effect. Reducing sample size in this manner is an important component of ensuring adherence to the three R’s, namely Replacement, Reduction, and Refinement, the principles that guide humane animal research. We will hope to further convince the reviewer of how sources of variation were controlled for in responding to subsequent comments.

2. This manuscript is twice as long as it needs to be. Almost every part of the manuscript could be reduced by ½ and in so doing would be come more focused and valuable.

We appreciate that the manuscript is long and will endeavour to reduce its length substantially in response to both reviewer comments.

Specific comments:

Title: Why are there 2 titles? Perhaps a requirement, but I prefer the 2nd shorter version.

This is a requirement of the journal.

Page 2, lines 38-40; The final statement here is an over interpretation of the data from a study with many sources of variation. The terms “imply” and “may” indicate that the authors likely feel the same way. Perhaps this study contains evidence that would support more research to better understand these last two points?

Respectfully, we do strongly feel that that our findings imply the safe use of dim red light at night over stabled horses for maintenance of normal sleep behaviour and we hope to convince the reviewer further of this within our rebuttal. However, we appreciate the direction on wording in relation to the finding related to wakefulness and have amended the final part of the abstract to read “*and provides evidence supporting further research to better understand the role of blue-enriched LED light at promoting increased wakefulness during daytime in stabled horses.”*

Page 3, lines 47-49; There are several places through here where greater specificity would improve the writing and strength of the paper. For example, be clearer about how welfare is compromised or what specifically changed. How is sleeping behavior influenced? Consider improving this specificity throughout.

Thank you. The specificity in these lines has now been improved with specific examples from the cited literature describing how welfare is compromised and how sleep behaviour is influenced.

Page 5, lines 102-114; This long discussion in the introduction is meant to support the value of measuring spontaneous blink rate in horses as an indicator of changes in dopamine transmission. While this connection may exist, it is not established in horses and jumping to using it as a response variable is questionable. I think this could still be included as a response variable, but with much less emphasis on what it my be connected to in regard to dopamine physiology.

Using dopamine agonist administration protocols, the link between elevated dopamine and blink rate has been tested in mammalian species with relatively primitive brains (rodents) (Kaminer et al., 2011) and more developed equivalents (non-human primates) (Kleven and Koek, 1996). The horse, falling between these two examples, is highly unlikely to exhibit differential characteristics in respect of dopamine and blink rate. However, we accept that to date, this has not been empirically tested. Therefore, we have shifted the emphasis away from dopamine transmission, over to what blink rate is telling us about the animal’s response to stress. In this regard, Mott and McBride (2020) recorded a significant positive correlation between salivary cortisol and blink rate in the horse (r = 0.56, p < 0.001), suggesting that blink rate can indeed be used as an indication of stress perception in this species. The introduction has been amended to reflect this change.

Page 5, lines 115-118; Consider listing a clear hypothesis.

Thank you. We have now added a clear hypothesis statement.

Page 6, line 126; As soon as any research is being conducted the procedures should be Category C. If this was categorized as B, that should be reviewed and rectified so as not to cause confusion.

We gained funding from the Morris Animal Foundation who, as part of the funding bid, ask extensively about the ethics and welfare of the study and for categorisation against USDA pain and distress categories. This information is scrutinised by their Animal Welfare Advisory Board and no concerns were raised about the use of the Category B description during this process. We refer the reviewer to the categorization resource <https://www.morrisanimalfoundation.org/sites/default/files/files/2018-12/USDA-Pain-and-Distress-Categories.pdf> . We referred to the procedures within our project as being within routine husbandry procedures including physical restraint and where we did not manipulate the environment in order to observe animal behaviour.

Page 7, line 149; Here and throughout an effort should be made to clearly delineate stalls, from section of the barn from the barn. Is Table 1 a table or a figure? It seems to be more a figure, but it leads to some lack of clarity as to how the sections of the barn were separated. Is the dark line that separates A from B an actual partition?

A ‘stall’ is referred to as a ‘stable’ in the UK. The individual stables were housed within an American barn style building. Edits have been made throughout to make this more clear in response to both reviewers. The reviewer is correct that Table 1 is a schematic and is more suitable to be labelled and included as a Figure, which has now been done. The dark line across the aisleway has been removed as there was no additional partition here. Wooden panel walls that did not extend completely to the ceiling divided the stables. This is detailed further down in the Methods.

Page 7, line 159; It is unclear why red light was needed all night in this experiment, particularly as this was not the main focus of the experiment. It would have been better to investigate this in a separate experiment.

We are grateful that the reviewer has highlighted this lack of clarity. The aim of this experiment was to compare a commercially available lighting system (a system the reviewer commended earlier as a “well researched and developed LED lighting system”) designed to optimise circadian rhythms in the horse to a standard lighting regime used in a busy equestrian centre. The Equilume system employs a dim red light continuously at night. This red light wavelength and intensity was previously shown to permit the nightly rise in melatonin (Murphy et al., 2019), a primary mediator of the circadian signal throughout the body. In a similar study conducted at a racehorse training facility where horses were frequently disturbed by light at night for feeding (Collery et al., 2023), this system stimulated strong 24-h rhythms in circadian clock genes that were absent (flat-lined) in horses under their normal lighting regime. The continuous exposure to red light at night prevents the disturbance to melatonin caused by erratic nighttime light, while permitting visibility for staff. However, the impact of red light at night on sleep behaviour had not previously been investigated and was in fact an important focus of the study. We have now emphasized this focus by extending the final sentence in this paragraph in the introduction (line 113) as follows: “These findings led to the development of a stable lighting system incorporating blue-enriched polychromatic white light by day and red light at night [36], *but the impact of this lighting system on sleep behaviour in horses has not yet been investigated*.” The reviewer’s suggestion of the inclusion of a clear hypothesis statement in the introduction should now also further clarify this focus.

Table 5; Is lateral recumbency sleep or awake? Based on later reading, I believe it to be categorized as sleep. For consistency and clarity, that should be listed here as it is for the other variables. In fact it would make sense to define each row with these terms.

Thank you. Each behaviour has now been categorised in the table as ‘sleep’ or ‘awake’, with the exception of unknown.

Page 15, lines 295-299; The large number of different individuals evaluating video footage is understandable and likely necessary. The use of agreement statistics to evaluate any vaiability or concerns here is good. However, the discussion of the lead author spot checking and a lack of description of how early training and calibration of students was performed leads to some concerns as to the additional noise that may be in this data.

Thank you. In these lines we state that the lead author checked to confirm that the time durations of all behaviours for each horse recorded by each observer summed to 24 h on each day. This is because each 24-h period required viewing of 20-30 video files (duration of each file roughly 45-50 minutes) within that timeframe and accounting for when each behaviour started and stopped to the second in a spreadsheet. Possibility for error of course existed in inputting exact durations of behaviours, but only accounted for the occasional loss of fractions of a minute which was corrected for during these checks.

For the benefit of the reviewer, three meetings were held with observers for training and calibration prior to evaluation of the experimental video footage. The first meeting was in-person and involved a detailed explanation of the ethogram with video examples of each behaviour followed by feedback and fine-tuning of the ethogram. A second online Zoom meeting introduced the sample video clips used during training and explained how to correctly record observations into the spreadsheets for evaluating inter-observer reliability. A final online Zoom meeting with observers provided feedback on the sample video clips and clarified any remaining questions the observers had before receiving the experimental videos for their respective horses. This final meeting occurred in the week before observers received access to their videos. A brief summary of these meetings and training is now included in this section to alleviate concerns about background noise.

Page 15, line 306; Please clarify the differential choice to use Shapiro Wilkes in one place and D’Agostino & Pearson in another for normality testing.

The Shapiro Wilks test is considered to be more powerful than D'Agostino-Pearson for smaller sample sizes. We re-ran the normality testing of SBR data using d’Agostino-Pearson, just to check, and it also returned a result of normality for the SBR data.

Page 15, lines 308-310; The description of ANOVA or Friedman’s here is unclear. What data was this conducted upon?What was the reasoning?

We thank the reviewer for highlighting this lack of clarity. There is indeed an omission in the description of how the data was analysed here that causes confusion. The first set of analyses utilising t-tests and Wilcoxon tests, depending on normality, analysed differences in the mean total durations for each behaviour parameter between treatment and control lighting conditions AND between Day and Night lighting periods and the results of these tests appear in Table 6 (now numbered Table 4). This description of the tests used for these analyses has now been corrected and the information re-ordered more logically.

The second set of analyses interrogated differences between duration of behaviour between the lighting periods defined as ‘Night’ and ‘Day’ both within and between the treatment groups using repeated measures ANOVA and or Friedmans (depending on normality) followed by multiple comparisons tests. These specific tests were required as they allow for assessment of three or more groups and we had the four groups: Control Night, Control Day, Treatment Night and Treatment Day and the results appear in Table 7 (now numbered Table 5). The Friedman test is a non-parametric alternative to the repeated measures ANOVA, used when comparing three or more related groups, but when the assumptions for a one-way ANOVA are not met. In this case, it was used only where data was found to be not normally distributed.

More details of the models used should be listed. A better description of the model will be important in fully interpreting tables 6 and 7.

Additional details about the use of these tests are now listed to improve clarity and interpretation of Tables 6 and 7 (now numbered 4 and 5).

“repeated measures” does not need to be abbreviated.

We have removed this abbreviation throughout.

Page 16, lines 337-341; This section on ICC and agreement should really be listed in the materials and methods.

Thank you. We have moved this paragraph to the relevant section in the methods

Page 17, lines 343-345; The primary sentence her that describes table 6 is unclear. Consider rewording.

Thank you. We have carefully reworded this sentence to improve clarity.

Table 6; In this table, the extra time spent out corresponds to extra time spent standing in the stall. These are both in the treatment group and the closest to “significance”. This simply highlights potential source of variation that are outside the primary aims of the experiment.

Respectfully, Table 6 (now 4) demonstrates the absence of any statistical difference in time spent ‘out of stable’ and the time spent ‘standing’ between the two lighting conditions. It is unclear what the reviewer means by saying that they “are both in the treatment group” as the P value relates to the mean differences between the two lighting conditions and neither could even be considered a trend given that both are >0.1. On the contrary, this finding indicated that there was less variation than could have been expected in ten horses kept for use within a busy equestrian centre.

Table 6; Why is there so much missing data at night? Much more than the day!

This is because at night there was no illumination of the barn aisleway, making it more difficult to identify behaviours when horses had their heads over the door and facing away from the position of the camera.

Table 7; It is curious that the p-value for the comparison of total recumbency during the day of 458 vs 18 is >0.999, while many other p-values are much lower. Is this correct?

Yes, this is correct and can be explained by considering that the units are in seconds and a thousand fold lower by day than by night (for treatment). Further, the variation around the mean is very high for daytime values compared to nighttime values. This is because, as illustrated in Fig 8A, only 5 horses under the control condition lay down during daytime, and only one of the horses became recumbent during daytime under the treatment lighting.

Page 21, line 390; The results here need to be more clearly described. Ultimately, there were day vs night differences. Further is a question of whether there was any interaction between lighting condition and day or night. The way this is written could easily be misinterpreted to indicate that the lighting condition is a “part” of the night versus day effect.

Thank you. This has now been reworded for improved clarity here and in subsequent results sections.

Page 23, line 448; The lack of a treatment effect in this study is not completely surprising, mostly due to the poor control of so many other sources of variation. At the end of the day, this is a poorly setup experiment, so positive or negative conclusions are very difficult to draw.

We respectfully disagree with the Reviewer that this was a poorly set-up experiment considering the nature and power associated with a crossover design, the effort employed to ensure that each experimental animal received each lighting treatment within their allocated stable without disruption between lighting treatments, the 4-week time period of each treatment, the lack of difference in mean age between groups and the regularity of the daily routine throughout the experiment. The finding that there was no difference in the amount of time horses spent out of their stables under each treatment was fortuitous and further strengthened the findings by reducing a potential variable that was out of the researchers control. The 30-min SBR recording sessions were conducted during the quietest time in the daily barn regime and lack of significant differences in blink rates may be explained by the similar management of all horses.

Page 24, lines 467-479; Far too much discussion of a non-significant difference. Delete all of this.

The statement referring to the non-significant finding of increased recumbency by day in the control condition has been removed. The rest of this paragraph related to the significant Day/Night difference in wakefulness in the treatment condition only. The discussion of this finding is warranted as this is the only behaviour that did not also show a day/night difference in the Control condition. These are the same group of horses responding differently to the Control lighting and a short discussion of why this might be is justified. In light of the reviewer’s objection, we have however reduced the discussion of this point substantially within the manuscript.

Page 25, lines 486-488; This is an interesting point and worth attention, particularly to others that may be developing or planning similar work.

Thank you. We have given it further attention by explaining why greater clarity might be needed.

Page 25, lines 489-490; While nonsignificant, time outside the stable was on contrast that came closest to significance. Feels a bit as if results are being cherry-picked only if they fit the theories of the authors. In lines 491-492, details are give about activities that were not consistently recorded. This is problematic as it is impossible to really analyze.

In light of these comments and the recommendation of Reviewer 1, this entire paragraph has been removed.

Pages 25-26, lines 499-510; Not critical, but again, information that could be helpful to others attempting to complete this kind of work.

Thank you. We have kept this information within the manuscript as we agree that it could be helpful to future research in this area.

Page 26, line 512; “does not negatively influence” is an awkward double negative that may lead to an unclear interpretation.

This has been replaced with ‘impact’.

The attention to and reporting of a lack of effect of redlight at night should be removed from the manuscript. It was not a primary objective, and the experimental design does not allow for this analysis appropriately. Really a separate experiment should be conducted. At best, this should be a brief mention.

We thank the reviewer for highlighting the lack of clarity in adequately describing the objectives of this study. These indeed included assessing whether the dim red light at night impacted sleep behaviour as part of the customised LED light system that was compared against a commonly used lighting regime in an equestrian facility.

The research that led to the development of a stable lighting system designed to strengthen circadian rhythms in horses is described in detail in the Introduction. This includes both the melatonin suppressing role of blue-enriched light by day and the melatonin protecting effects of dim red light at night for horses. In response to an earlier comment by this Reviewer, we also now highlight how the impact of this red light has not previously been evaluated for its impact on sleep behaviour in horses. This addition, combined with Reviewer 1’s request to include additional information in the Introduction on the benefits of red light for maintaining rest-activity behaviour patterns in other species, should now better emphasize the importance of this aspect of the experimental objectives. In addition, the inclusion of a clear hypothesis statement at the recommendation of this Reviewer should further enhance the understanding of the reader.

We suggest that the data analyses, especially the multiple comparisons of Treatment Night, Treatment Day, Control Night and Control Day does in fact allow comparison of behaviours between darkness or dim red light at night.

Notwithstanding the above, and in response to the reviewer we have significantly reduced this paragraph to more briefly mention this point.

Page 27, line 530; Rewording required.

This has been done.

Page 27, lines 537-543; This paragraph should be deleted. This analysis goes beyond what the data presented can support.

This paragraph has been removed.

Pages 27-28, lines 544-563; Delete this paragraph. It is not supported by the results of this study and detracts from a clear and concise manuscript.

We have removed the first half of this paragraph as well as the final sentence. However, the remainder highlights and explains an important difference in results between this study and a similar study in stabled racehorses. It highlights the variability in lighting regimes that horses are kept under and the impact this has on their physiology and behaviour and we feel its inclusion is justified.

Page 28, line 576; “quasi-experimental nature of this study” highlights the major weakness of this study.

The term ‘quasi-experimental’ was inappropriately used here to refer to the fact that the horses used in the study continued with their normal regime as working animals in an equestrian centre, in contrast to animals maintained at a dedicated research facility. This aligned with the overarching goal of the study to shed light on the impact of standard lighting regimes on horses in the industry that could be most relevant to inform industry practice and to potentially improve conditions. The strong design of the experiment wherein each animal experienced each treatment and acted as its own control allowed for a significant reduction in sources of variability and the behaviour and functional genomics data produced was supported by training and statistical agreement of observer reporting and rigorous laboratory techniques respectively. This sentence has been more appropriately rephrased as follows *“Due to the objectives of this study, such that the impacts of lighting were assessed in horses housed in their usual stables on a working yard..”*

There is value in what was done here and the data/results should be presented in a much more clear and concise manner for others to use in designing future research in this area.

Thank you, the comments of both reviewers have contributed to a more clear and concise manuscript.

Page 29, lines 596-597; Due to the fact that there is not a clear definition of what “circadian health” is, id don’t think that the data from this experiment support this statement. The horses in this study were not clearly stressed, but their health and well-being is not 100% clear. Mightn’t they have been healthier if they had more time in paddocks? Just unknown.

We have removed this sentence.

Page 29, lines 598-600; None of this is really supported by the findings of this study, particularly any potential benefit of dim red light. More work is needed to support this.

We respectfully disagree and have previously responded on this point and amended the manuscript to reflect reviewer concerns and recommendations throughout.

Page 29, lines 602-603; A comparison of wakefulness between the treatment and controlled groups during the day indicates no difference. It is the day vs night difference that is significant. This feels like it is being twisted to support the authors’ hypothesis that the treatment is better.

We have previously responded on this point above. However, we have now removed this sentence from the conclusions.

References; There are too many references. The authors should be more selective. This is not a literature review.

Thank you. The reference list has been reduced following the removal of numerous paragraphs in the discussion. We have also now been more selective throughout where multiple references were used in support of a statement.

Figure 10; This figure could be improved by bringing the graphs closer together and using a common x-axis.

Thank you. We have moved the graphs closer and used a common x and y axis.

General comments:
1. There are a number of sources of variation that could have easily been avoided and are disappointing to see. I will record some of those here. The LED lighting system was on a timer, but the control system was turned on and off manually, with greater variation.

This was as planned. We wanted to evaluate a normal lighting regime used in a working barn as per staff requirements against a controlled system.

Some horses were blanketed overnight and some were not and it is unclear who and when and if this had any effect.

This was controlled for in the crossover design. The management (including feeding/blanketing/weekly frequency of use for lessons/demos) for each horse did not change over the duration of the study and each horse acted as its own control and experienced both lighting conditions. If a horse normally wore a blanket at night, this was maintained through both experimental lighting periods.

One horse was fed concentrate meals while the others were not. Another source of variation. The exercise regimes and time out of the stable further contributes to variation.

Both of these factors were controlled for in the study design. One horse required concentrate to maintain a healthy body condition and this did not change throughout the experimental periods for this animal. The exercise regimes may have differed slightly between horses, but each horse maintained a similar weekly schedule throughout the study. This is supported by the lack of difference in time spent out of the stable under each condition.

There is lack of clarity as to how this was accounted for in the statistical analysis.

It was not accounted for in the statistical analysis. It was accounted for in the overall study design.

There is some talk of the “spillover” of light from the sections or stalls of the barn. The authors seem to indicate that this is not a large concern, and yet their research and much of the other research in this area, that a small exposure to other wavelengths can have a significant effect. All these things combined represent a significant concern in the interpretation of findings from this study.

Light intensity and wavelength readings were taken during Day and Night from all stalls under each lighting condition. As can be seen from the data presented in Table 1, there is minimal variation around the mean light intensity in each condition and lighting period. The reviewer’s concern would be warranted if any of the control stables had recorded a lux reading greater than 1 at night. Wavelengths of light previously shown to impact horses occur at >10 lux. The inclusion of this point as a limitation was an artifact of the thesis written by the student whose Masters project this was (E. Harkin), wherein she was strongly advised to list all potential limitations of the study. Subsequently, the analysis of the recorded light intensity data from each stable highlighted that this was not in fact a concern. Therefore, we have removed this paragraph from this section as it is already sufficiently addressed in the Methods section.

2. One of the major highlighted findings is the “higher wakefulness during the day in the LED group”. This comparison is somewhat misleading as the difference really seems to lie in the fact that there was lower wakefulness recorded in the LED group at night. This difference does not translate into significant differences in sleep or recumbency. Every effort should be made to more clearly communicate and not potentially mislead here.

Every effort has now been made to correct any statements that may have mislead the reader. The finding that is highlighted is the significant day/night difference in wakefulness durations recorded in the 10 horses under the treatment condition when compared to the absence of same for the control condition, as this represents the only behaviour where a day/night difference was not detected in the control condition. Further, the reviewers comment that this finding is likely attributed to lower wakefulness recorded in the LED group is presumably from reviewing the mean values in Table 6 (now 4). However, the individual horse data for wakefulness presented in Fig 8 clearly shows that 8/10 horses demonstrated higher wakefulness durations under treatment conditions which provides better insight than a mean value.

3. In the statistical analysis section of the materials and methods, consider not using an entire sentence to describe the software. The analysis performed is most important and the software and version used should simply be listed in parentheses.

This change has been made, but please note that this change now necessitates the use of nested parentheses to indicate address of software manufacturer.

4. There are different perspectives on my point here; therefore, a suggestion. Remove the term “significant” except for where it is defined in the statistics section. If anything is deemed “different” or larger or smaller, one would assume that the difference reported was significant, or it was undetectable. In the same vein, either list specific p-values or no p-values, but listing < or >0.05 throughout is not particularly helpful. I would lean towards listing specific p-values.

Thank you. We have taken the reviewer’s recommendation to remove the word ‘significant’ throughout the results section. We have now also listed specific P values throughout.

5. Tables 6 and 7 are foundational to this manuscript, but the differences and reasons to have both tables is unclear. Clearly there were different statistical analyses conducted for each table. Why is table 7 alone not sufficient? It becomes particularly challenging when trying to compare the numbers in the 2 tables, as they don’t really match up, for example total recumbency. The multiple comparisons in table 7 are kind of like an interaction from an ANOVA? I am concerned that these individual comparisons and a lack of a clear correction for these multiple comparisons can lead to an increased risk of Type I errors.

We have addressed the different analyses and their presentation in these tables in a previous comment and believe that the inclusion of both is required for a full understanding of the data and interpretation of results.

6. There are too many figures illustrating individual horse data from this study. Particularly because most of them are illustrating data without differences. Consider rethinking the most important figures or table that must be presented. I think the number could be reduced by ½ to 2/3rds. There is also not a clear reason to have individual horse data in all of the figures.

Respectfully, given the number of parameters measured, we had already carefully selected the figures that we felt described the most important parameters and/or findings. However, for further reduction and simplification, we have merged Figures 3 and 5 to a Single Figure for SBR (now Fig 5). We have also merged Figure 7 and Figure 8 (now Fig 7) and on the reviewer’s earlier recommendation brought the graphs closer together and used a common x-axis. We have similarly amended the other results graphs to reduce their size. We respectfully feel that presenting the individual horse data for the main parameters of interest - sleep, recumbency and wakefulness – highlights the importance of understanding individual variation that will be informative for researchers in this field. The individual data also gives greater resolution of the data for meaningful interpretation of findings.

7. The general comment #1 above and various points through the paper lead to the conclusion that lack of control is a serious weakness for this manuscript. A potential way forward, to facilitate communication of the data and knowledge captured with this effort, would be to simplify the presentation of data considerably. Focus on table 6 or perhaps table 7 and go no further. Perhaps even a short communication that could be used to spur further research.

Respectfully, we have now made extensive amendments throughout the manuscript serving to improve clarity, reduce content, and simplify findings in light of both reviewers’ comments. We hope that we have now provided better clarity around the inherent strength of the study design.

6. PLOS authors have the option to publish the peer review history of their article ([what does this mean?](https://track.editorialmanager.com/CL0/https:%2F%2Fjournals.plos.org%2Fplosone%2Fs%2Feditorial-and-peer-review-process%23loc-peer-review-history/1/010f019543540d38-b37e0b64-93a7-4be1-b277-9b296f535f26-000000/Z92wlvqT6Hoebh7oKy1W2RGB55XX16oqo5GAFgpefXQ=199)). If published, this will include your full peer review and any attached files.


If you choose “no”, your identity will remain anonymous but your review may still be made public.


**Do you want your identity to be public for this peer review?** For information about this choice, including consent withdrawal, please see our [Privacy Policy](https://track.editorialmanager.com/CL0/https:%2F%2Fwww.plos.org%2Fprivacy-policy/1/010f019543540d38-b37e0b64-93a7-4be1-b277-9b296f535f26-000000/qTQhP0YiwFfMM-OaF2j9U7De9_XI4A_E7VWYe09eYJI=199).

Reviewer #1: No

Reviewer #2: No
